# Supplementary figures and images for: MYC overrides HIF-1α to regulate proliferating primary cell metabolism in hypoxia
Source: eLife. 2023 Jul 10;12:e82597. doi: 10.7554/eLife.82597 (PMC10332812; doi:10.7554/eLife.82597)

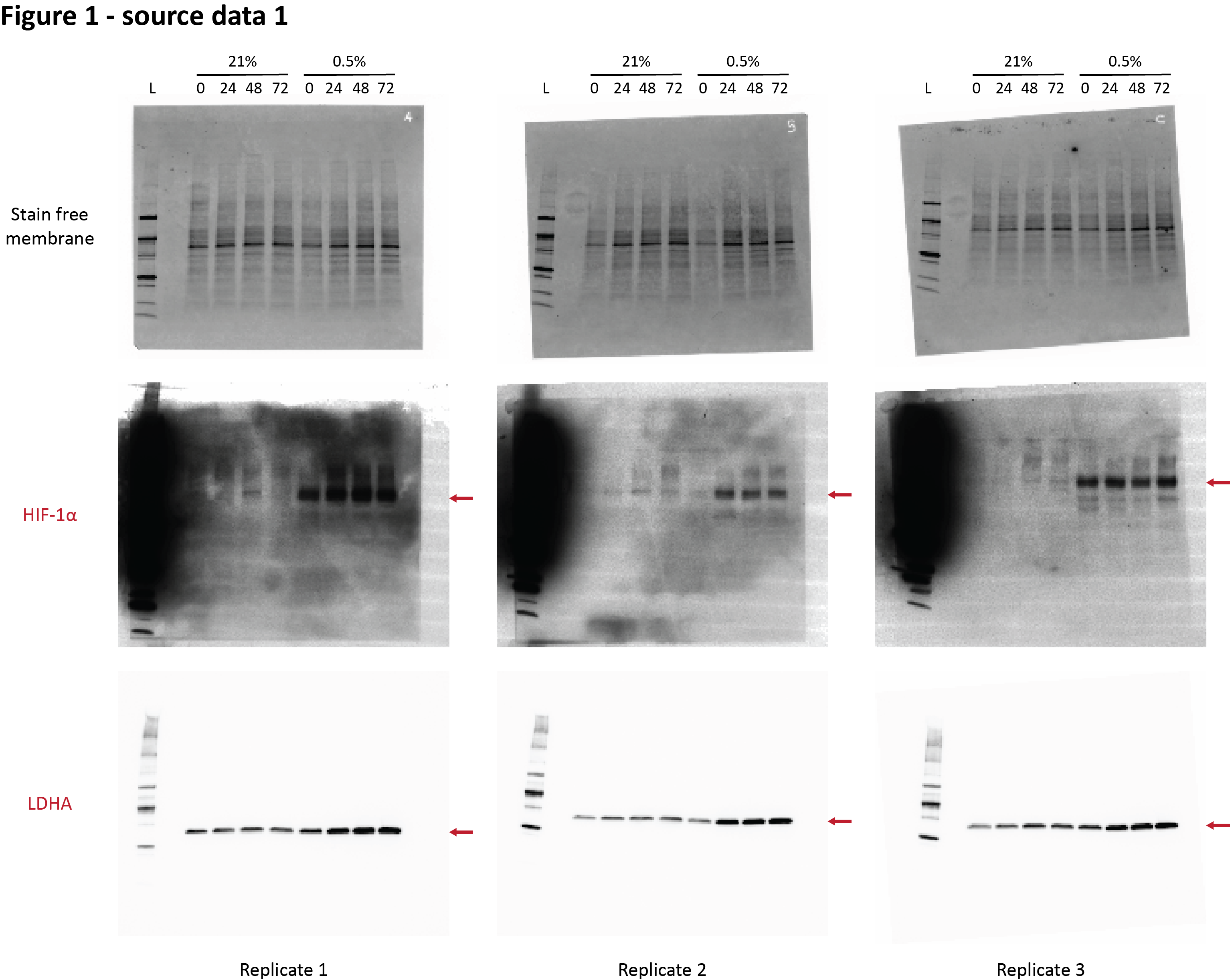

Supplement: Figure 1—source data 1. [file elife-82597-fig1-data1.zip › Figure 1 - source data 1.png]

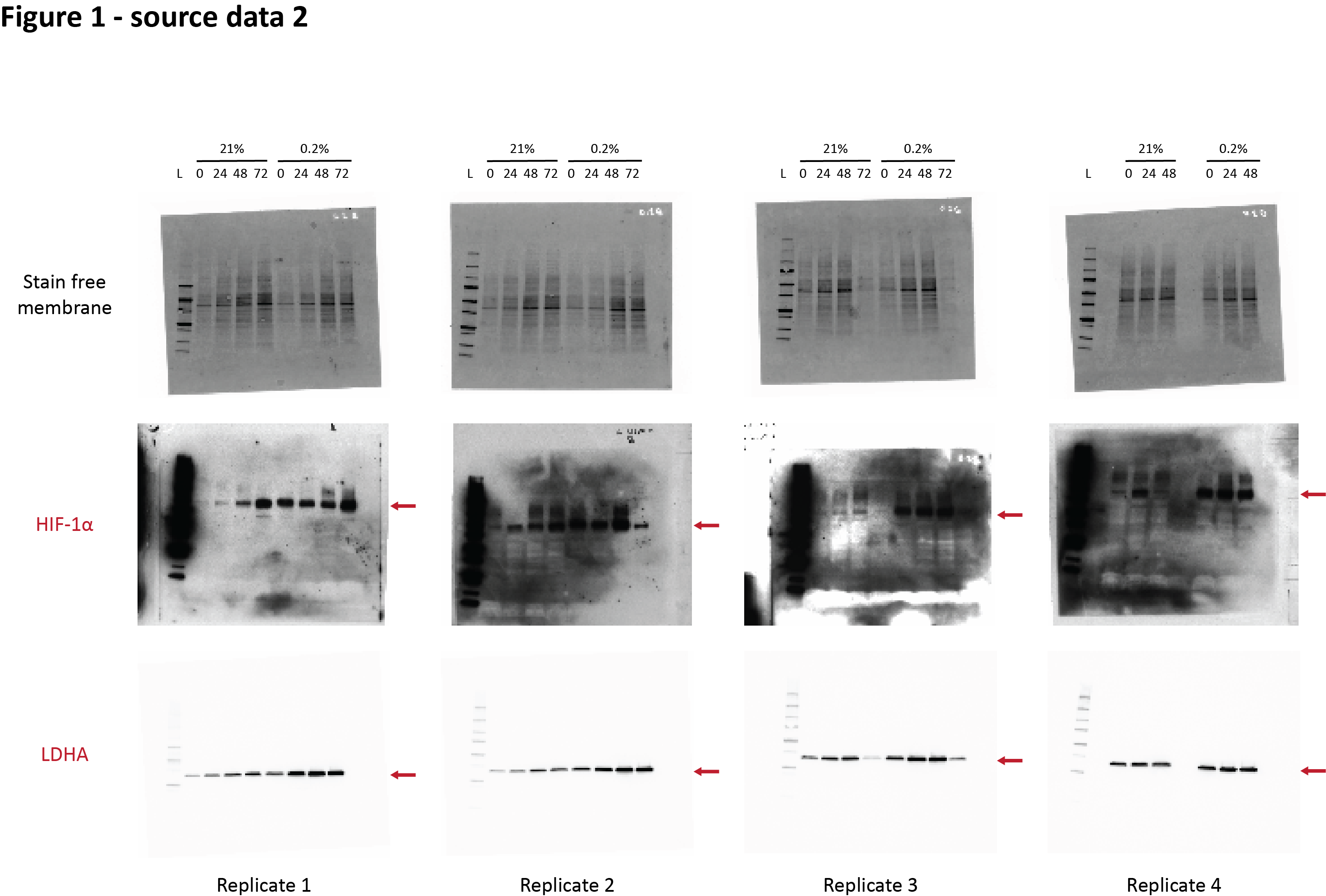

Supplement: Figure 1—figure supplement 3—source data 1. [file elife-82597-fig1-figsupp3-data1.zip › Figure 1 - source data 2.png]

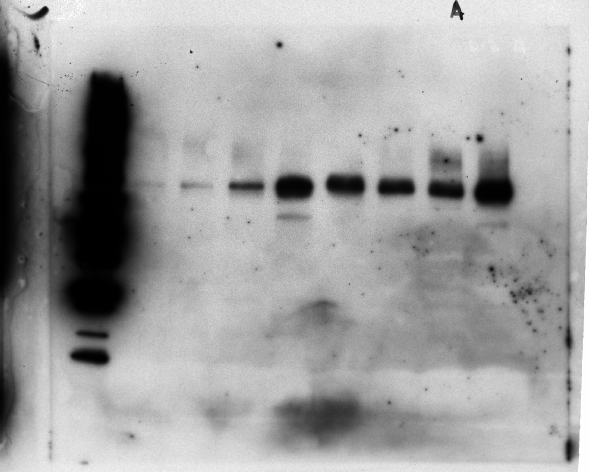

Supplement: Figure 1—figure supplement 3—source data 1. [file elife-82597-fig1-figsupp3-data1.zip › lf_02_a_hif1a_2018-04-20.tif]

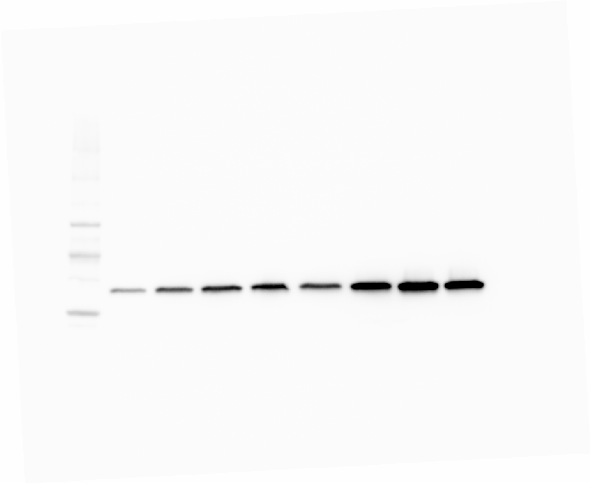

Supplement: Figure 1—figure supplement 3—source data 1. [file elife-82597-fig1-figsupp3-data1.zip › lf_02_a_ldha_2018-04-21.tif]

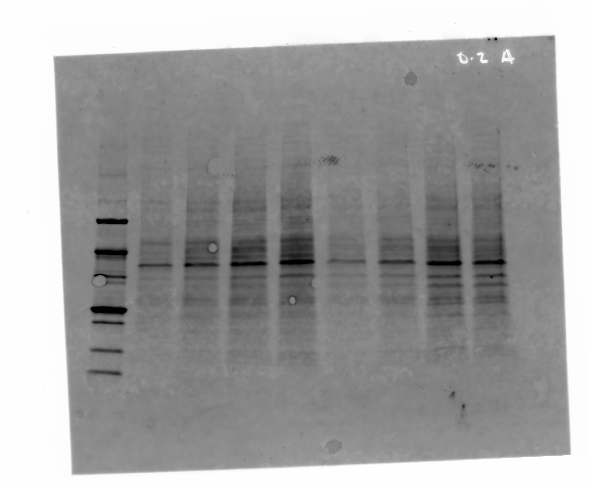

Supplement: Figure 1—figure supplement 3—source data 1. [file elife-82597-fig1-figsupp3-data1.zip › lf_02_a_membrane_2018-04-19.tif]

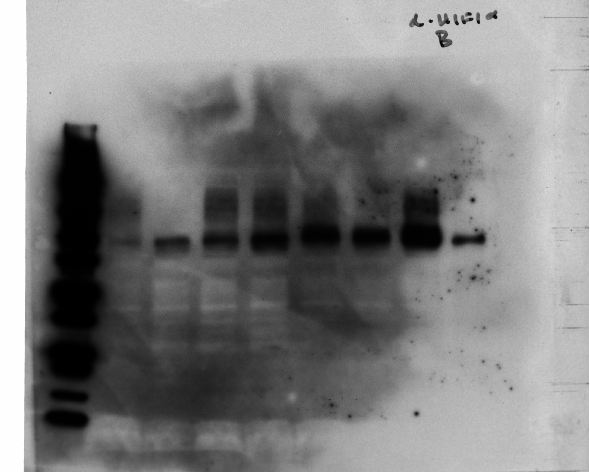

Supplement: Figure 1—figure supplement 3—source data 1. [file elife-82597-fig1-figsupp3-data1.zip › lf_02_b_hif1a_2018-04-20.tif]

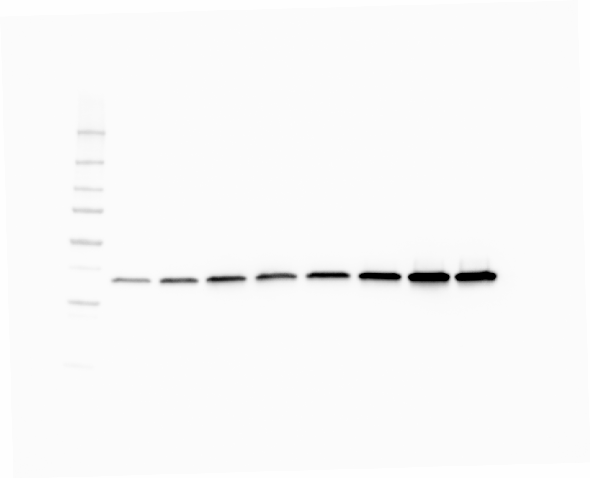

Supplement: Figure 1—figure supplement 3—source data 1. [file elife-82597-fig1-figsupp3-data1.zip › lf_02_b_ldha_2018-04-21.tif]

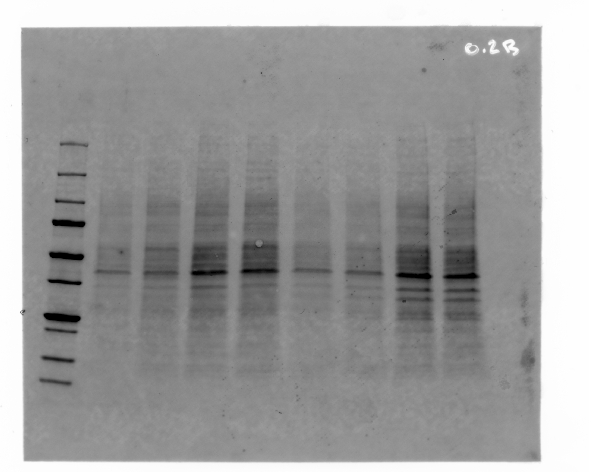

Supplement: Figure 1—figure supplement 3—source data 1. [file elife-82597-fig1-figsupp3-data1.zip › lf_02_b_membrane_2018-04-19.tif]

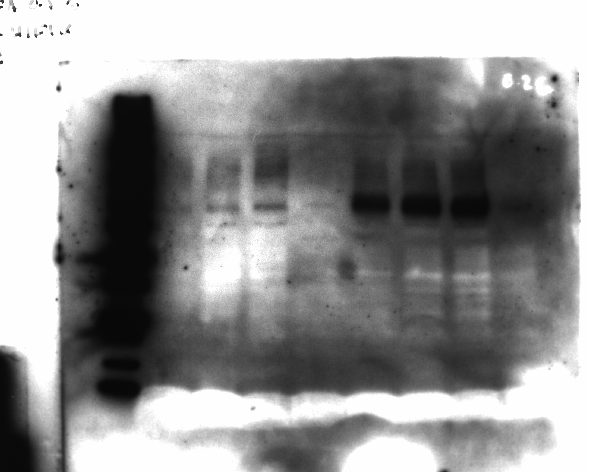

Supplement: Figure 1—figure supplement 3—source data 1. [file elife-82597-fig1-figsupp3-data1.zip › lf_02_c_hif1a_2018-04-20.tif]

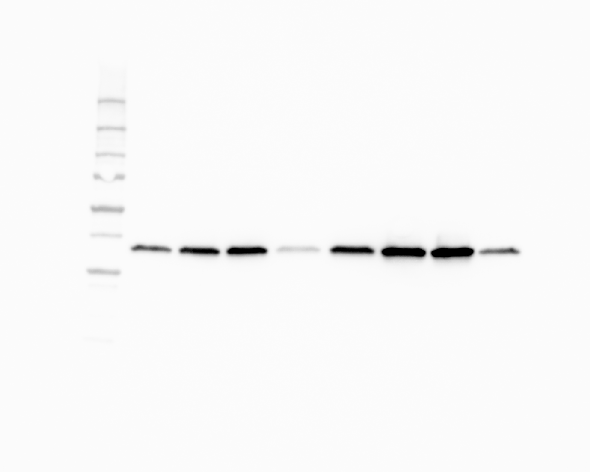

Supplement: Figure 1—figure supplement 3—source data 1. [file elife-82597-fig1-figsupp3-data1.zip › lf_02_c_ldha_2018-04-21.tif]

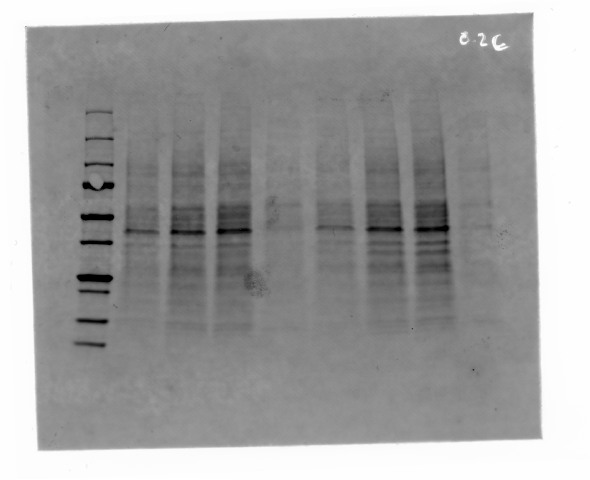

Supplement: Figure 1—figure supplement 3—source data 1. [file elife-82597-fig1-figsupp3-data1.zip › lf_02_c_membrane_2018-04-19.tif]

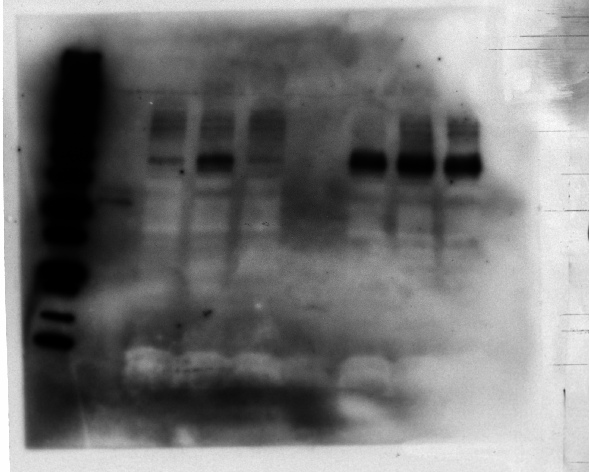

Supplement: Figure 1—figure supplement 3—source data 1. [file elife-82597-fig1-figsupp3-data1.zip › lf_02_d_hif1a_2018-04-20.tif]

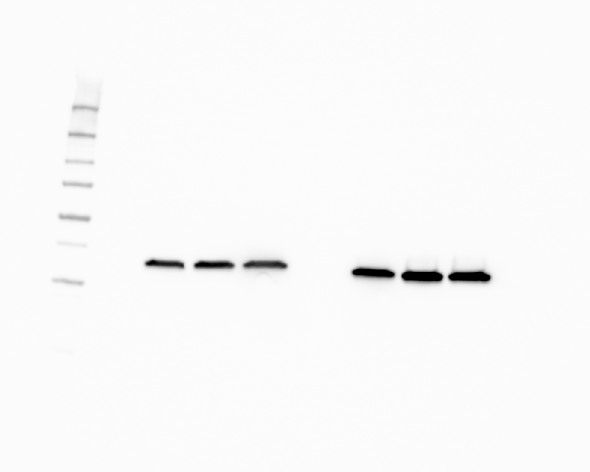

Supplement: Figure 1—figure supplement 3—source data 1. [file elife-82597-fig1-figsupp3-data1.zip › lf_02_d_ldha_2018-04-21.tif]

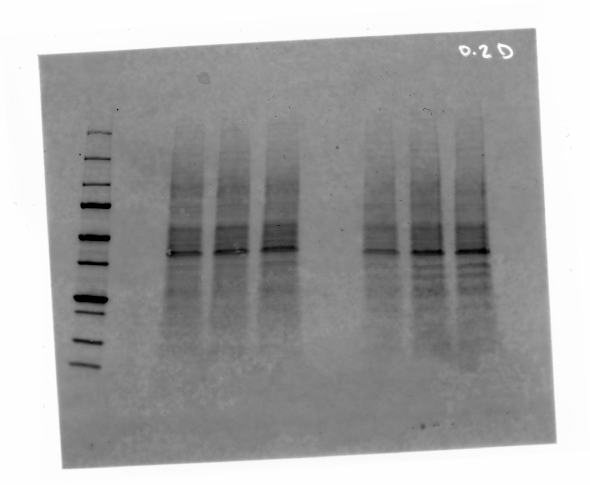

Supplement: Figure 1—figure supplement 3—source data 1. [file elife-82597-fig1-figsupp3-data1.zip › lf_02_d_membrane_2018-04-19.tif]

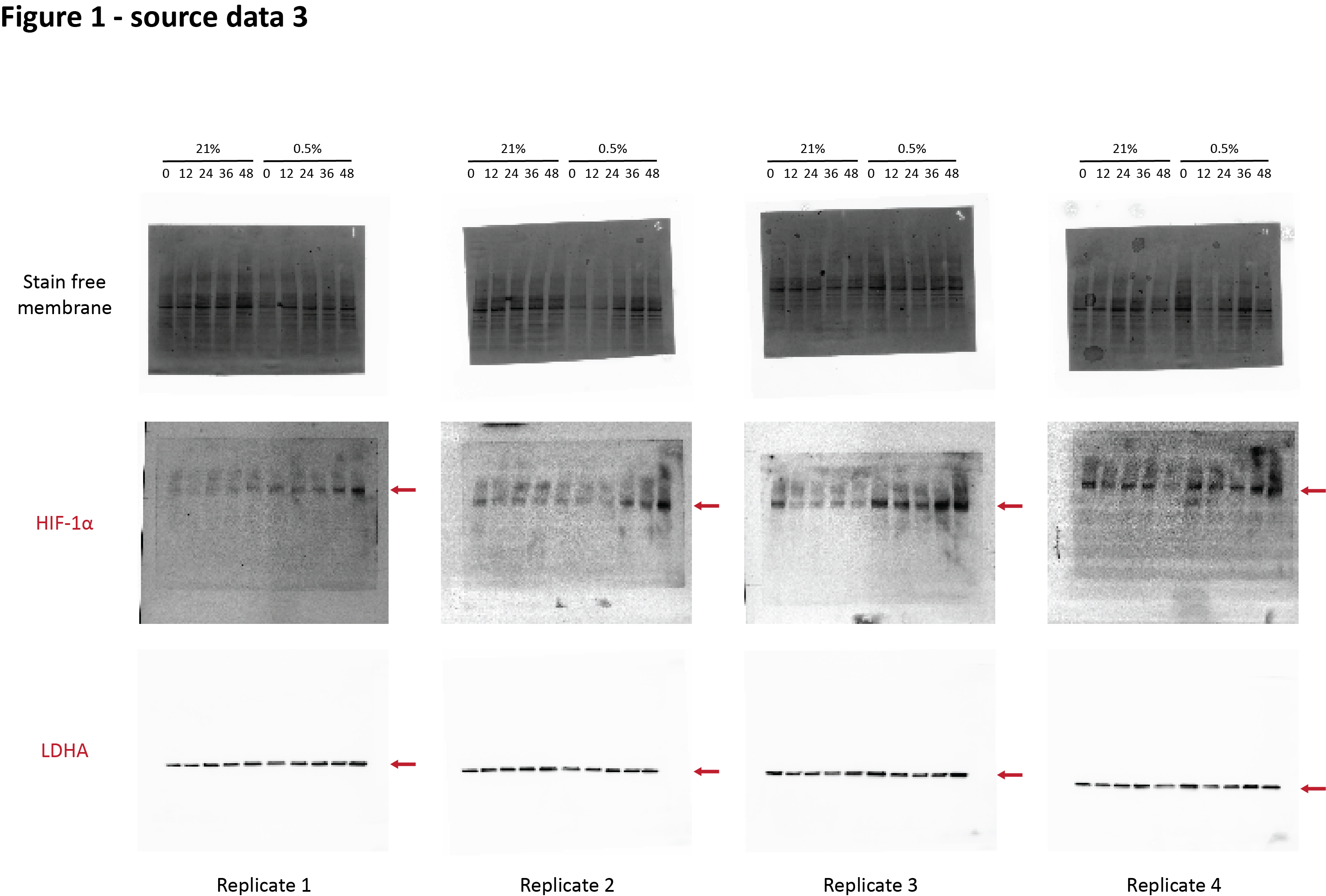

Supplement: Figure 1—figure supplement 4—source data 1. [file elife-82597-fig1-figsupp4-data1.zip › Figure 1 - source data 3.png]

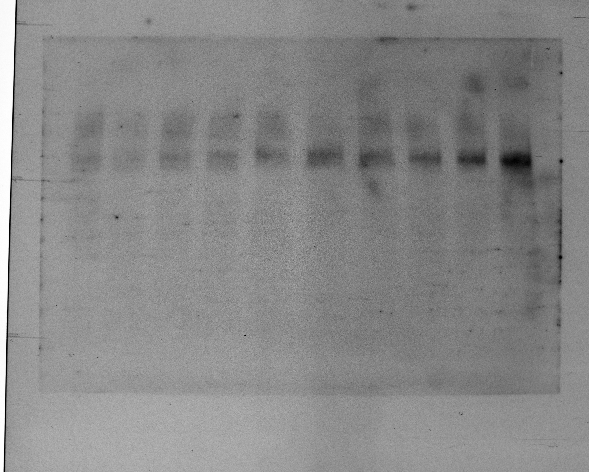

Supplement: Figure 1—figure supplement 4—source data 1. [file elife-82597-fig1-figsupp4-data1.zip › pasmc_05_a_hif1a_2_2020-10-08.tif]

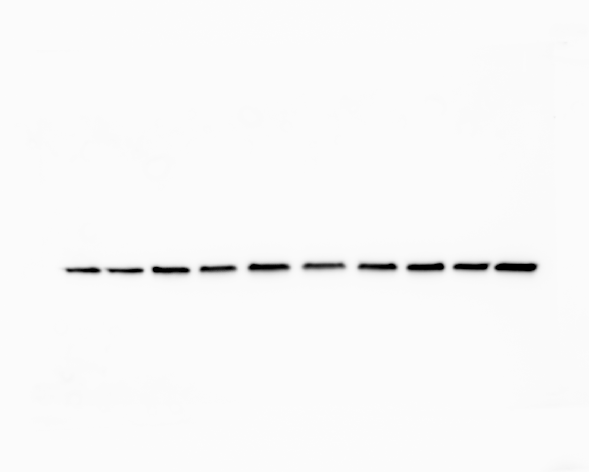

Supplement: Figure 1—figure supplement 4—source data 1. [file elife-82597-fig1-figsupp4-data1.zip › pasmc_05_a_ldha_2020-10-08.tif]

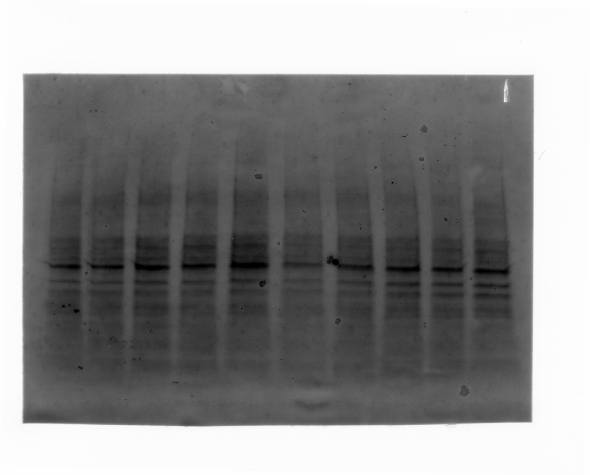

Supplement: Figure 1—figure supplement 4—source data 1. [file elife-82597-fig1-figsupp4-data1.zip › pasmc_05_a_membrane_2020-10-05.tif]

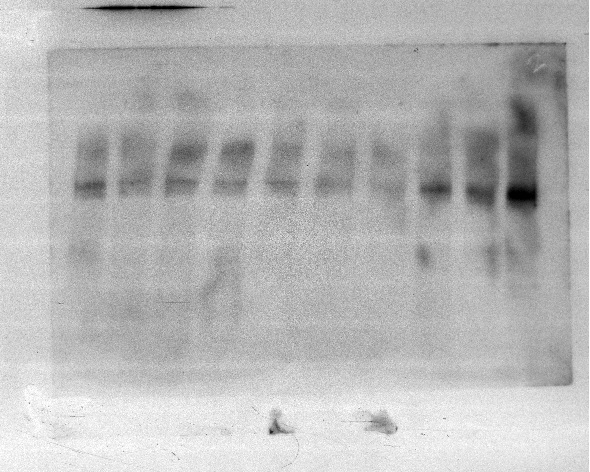

Supplement: Figure 1—figure supplement 4—source data 1. [file elife-82597-fig1-figsupp4-data1.zip › pasmc_05_b_hif1a_2_2020-10-08.tif]

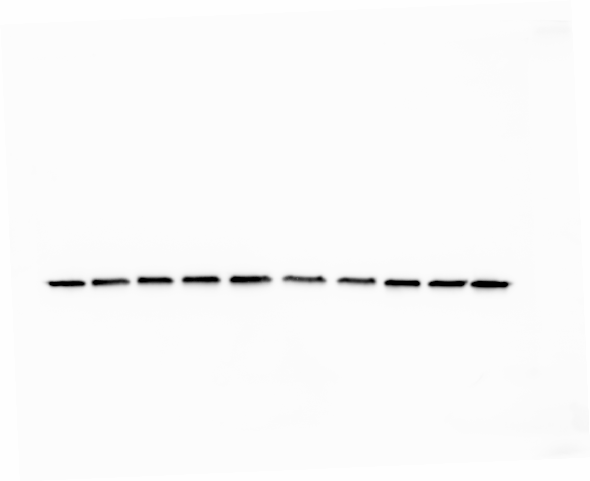

Supplement: Figure 1—figure supplement 4—source data 1. [file elife-82597-fig1-figsupp4-data1.zip › pasmc_05_b_ldha_2020-10-08.tif]

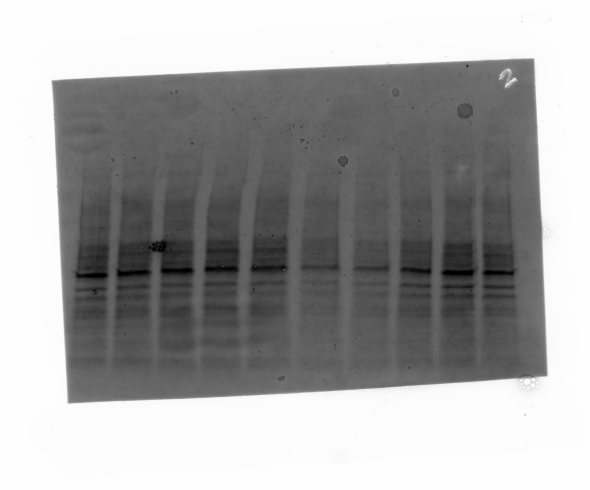

Supplement: Figure 1—figure supplement 4—source data 1. [file elife-82597-fig1-figsupp4-data1.zip › pasmc_05_b_membrane_2020-10-05.tif]

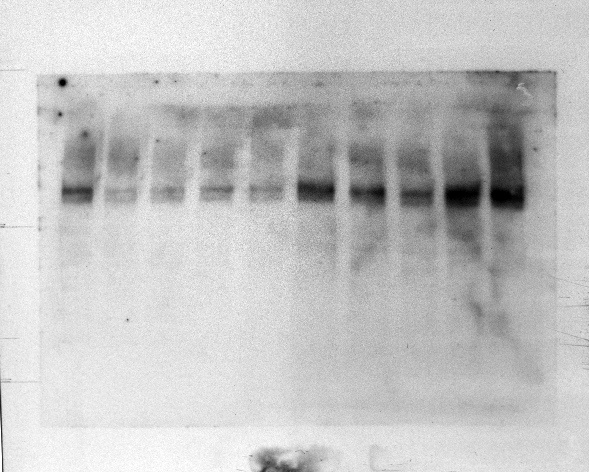

Supplement: Figure 1—figure supplement 4—source data 1. [file elife-82597-fig1-figsupp4-data1.zip › pasmc_05_c_hif1a_2_2020-10-08.tif]

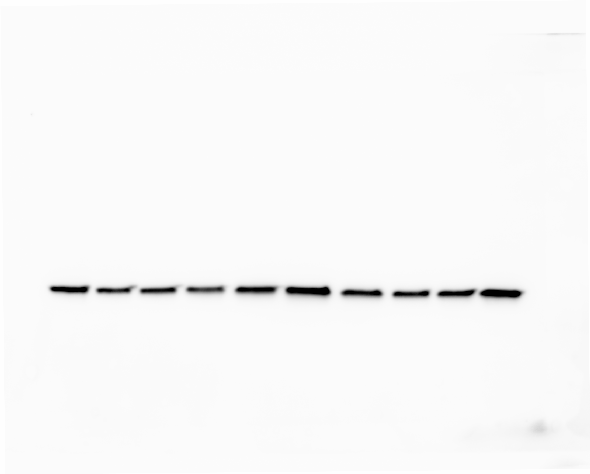

Supplement: Figure 1—figure supplement 4—source data 1. [file elife-82597-fig1-figsupp4-data1.zip › pasmc_05_c_ldha_2020-10-08.tif]

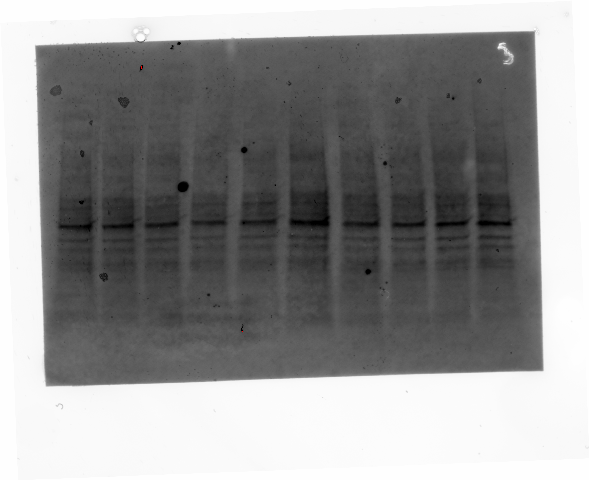

Supplement: Figure 1—figure supplement 4—source data 1. [file elife-82597-fig1-figsupp4-data1.zip › pasmc_05_c_membrane_2020-10-05.tif]

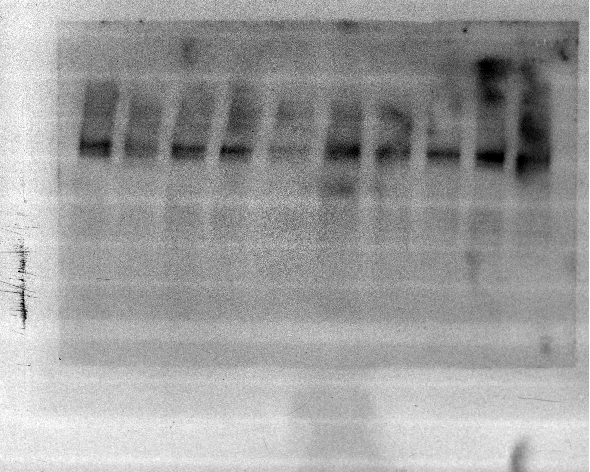

Supplement: Figure 1—figure supplement 4—source data 1. [file elife-82597-fig1-figsupp4-data1.zip › pasmc_05_d_hif1a_2_2020-10-08.tif]

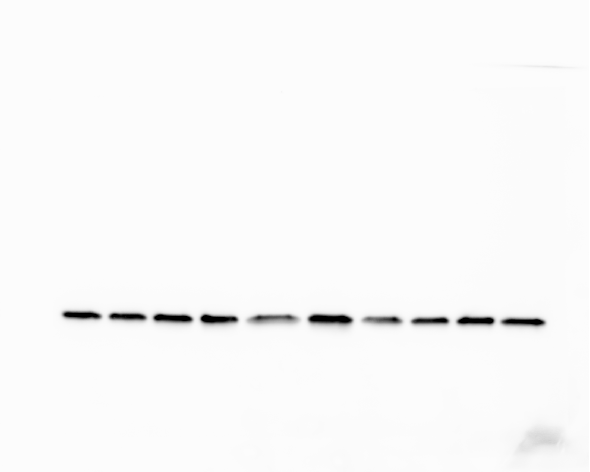

Supplement: Figure 1—figure supplement 4—source data 1. [file elife-82597-fig1-figsupp4-data1.zip › pasmc_05_d_ldha_2020-10-08.tif]

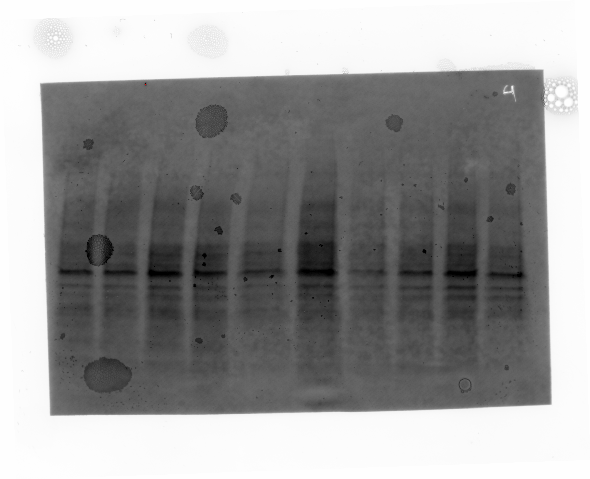

Supplement: Figure 1—figure supplement 4—source data 1. [file elife-82597-fig1-figsupp4-data1.zip › pasmc_05_d_membrane_2020-10-05.tif]

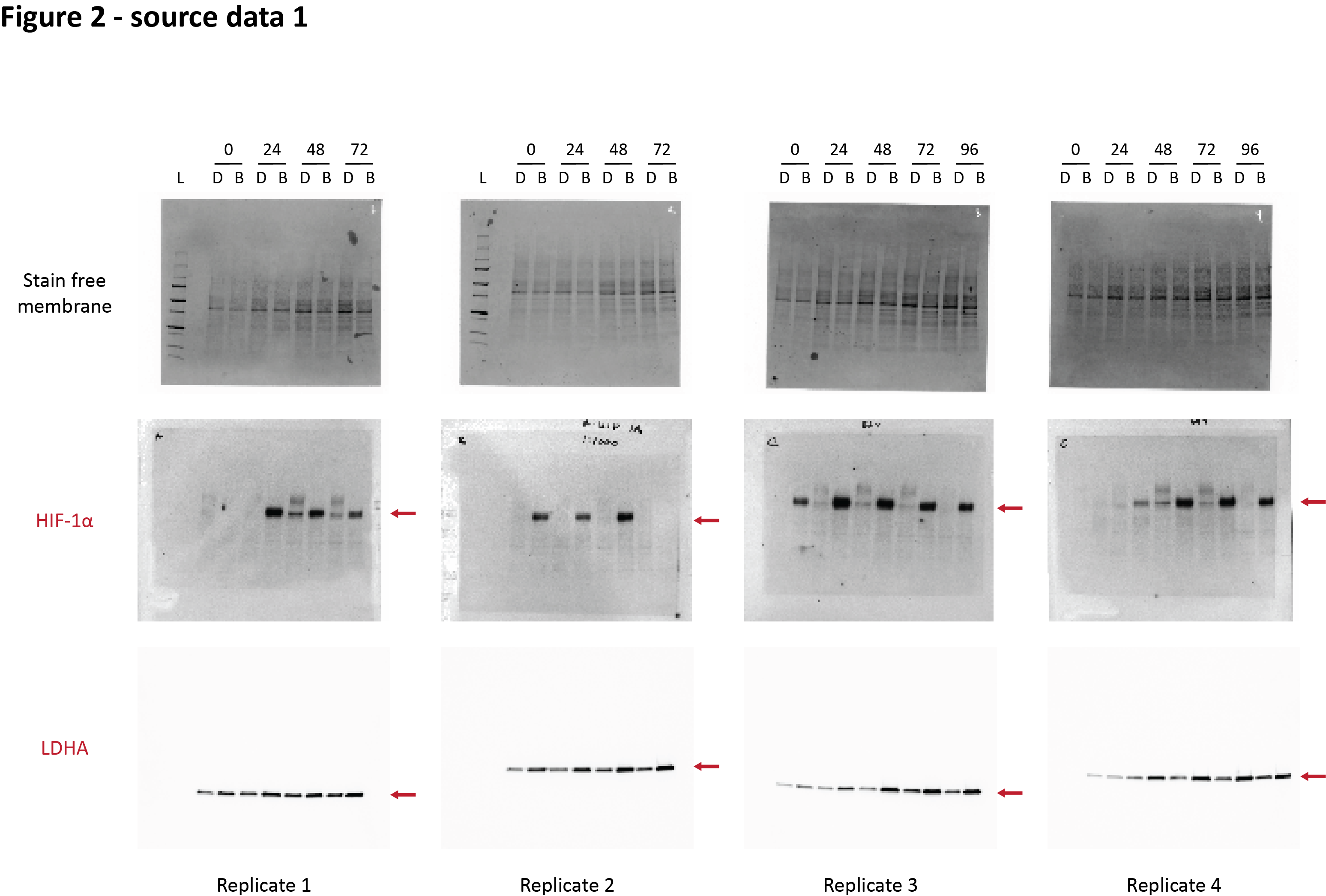

Supplement: Figure 2—source data 1. [file elife-82597-fig2-data1.zip › Figure 2 - source data 1.png]

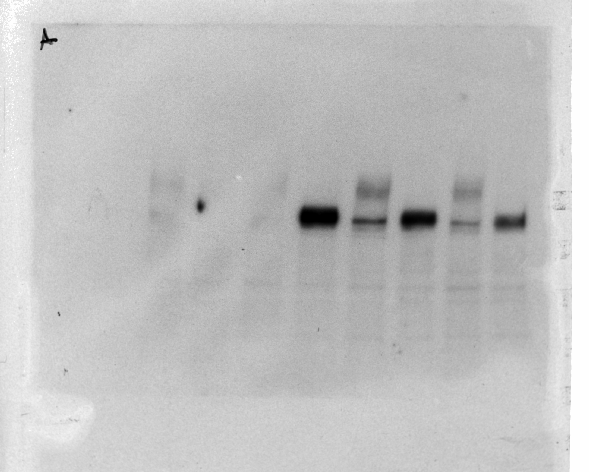

Supplement: Figure 2—source data 1. [file elife-82597-fig2-data1.zip › lf_bay_a_hif1a_2018-12-17.tif]

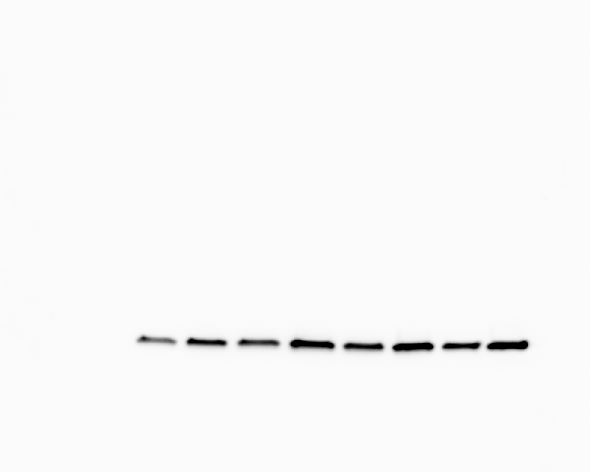

Supplement: Figure 2—source data 1. [file elife-82597-fig2-data1.zip › lf_bay_a_ldha_2018-12-17.tif]

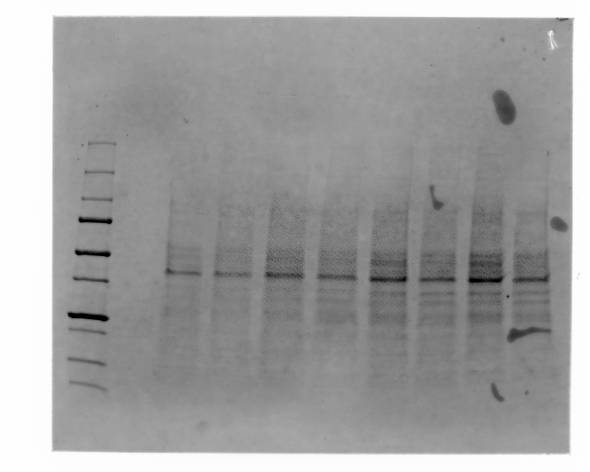

Supplement: Figure 2—source data 1. [file elife-82597-fig2-data1.zip › lf_bay_a_membrane_2018-12-17.tif]

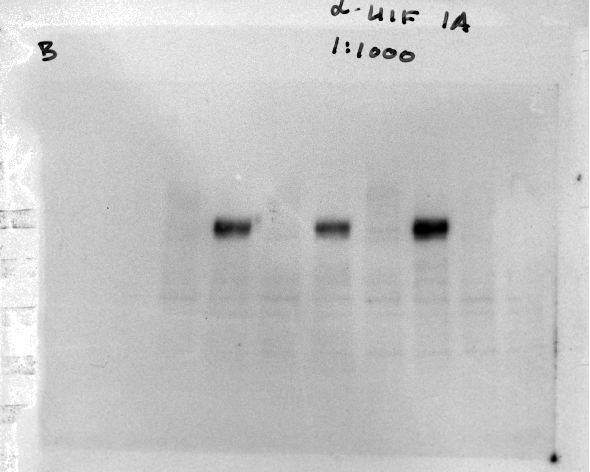

Supplement: Figure 2—source data 1. [file elife-82597-fig2-data1.zip › lf_bay_b_hif1a_2018-12-17.tif]

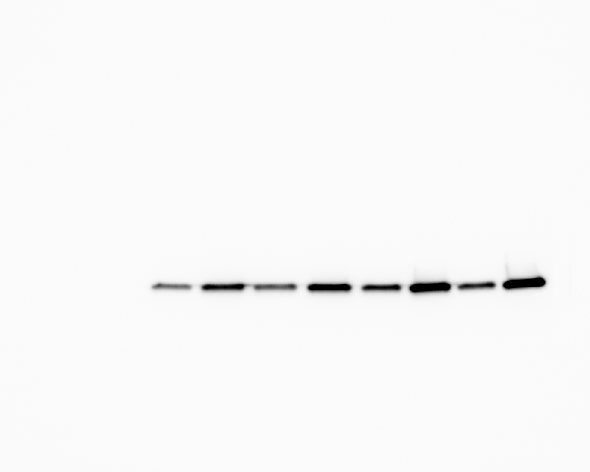

Supplement: Figure 2—source data 1. [file elife-82597-fig2-data1.zip › lf_bay_b_ldha_2018-12-17.tif]

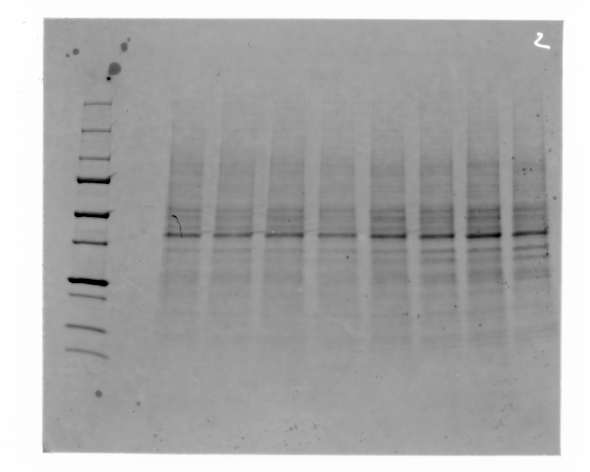

Supplement: Figure 2—source data 1. [file elife-82597-fig2-data1.zip › lf_bay_b_membrane_2018-12-17.tif]

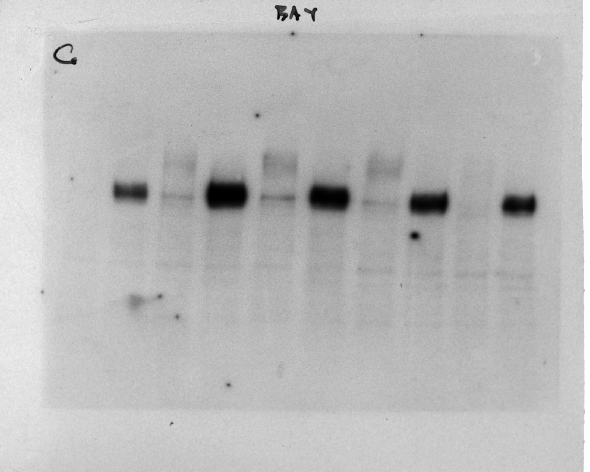

Supplement: Figure 2—source data 1. [file elife-82597-fig2-data1.zip › lf_bay_c_hif1a_2018-12-17.tif]

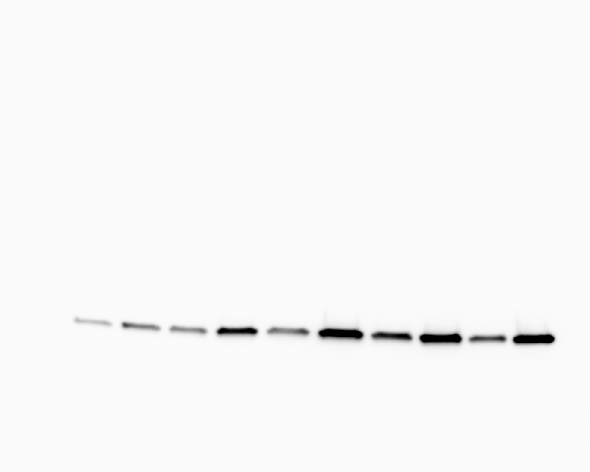

Supplement: Figure 2—source data 1. [file elife-82597-fig2-data1.zip › lf_bay_c_ldha_2018-12-17.tif]

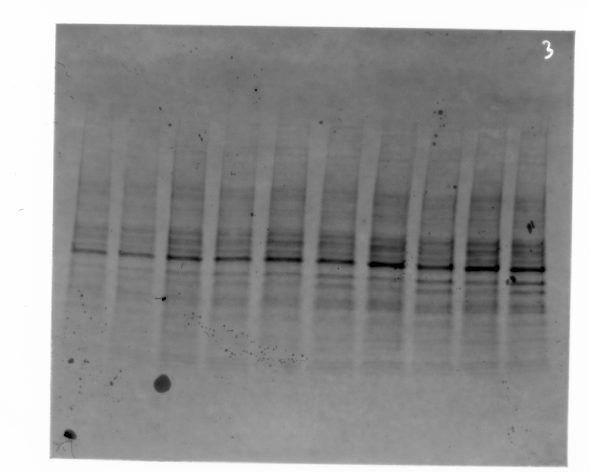

Supplement: Figure 2—source data 1. [file elife-82597-fig2-data1.zip › lf_bay_c_membrane_2018-12-17.tif]

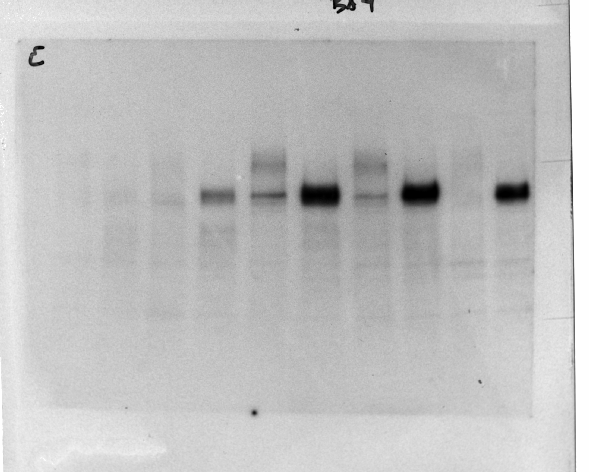

Supplement: Figure 2—source data 1. [file elife-82597-fig2-data1.zip › lf_bay_e_hif1a_2018-12-17.tif]

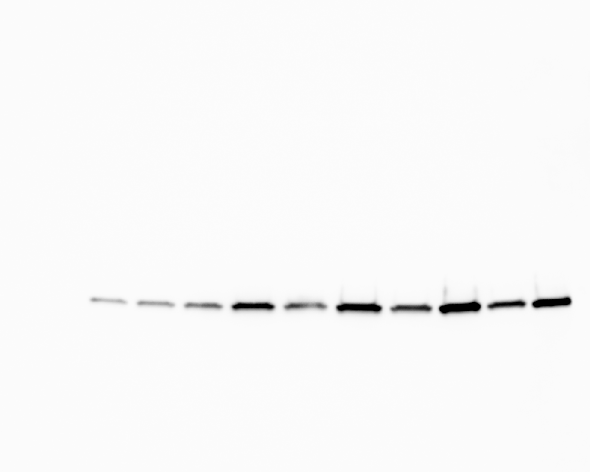

Supplement: Figure 2—source data 1. [file elife-82597-fig2-data1.zip › lf_bay_e_ldha_2018-12-17.tif]

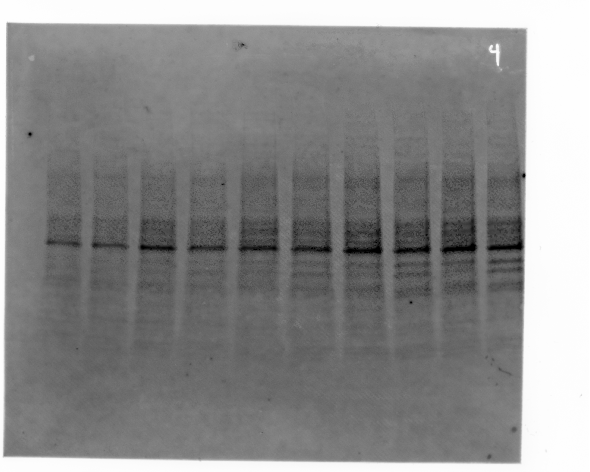

Supplement: Figure 2—source data 1. [file elife-82597-fig2-data1.zip › lf_bay_e_membrane_2018-12-17.tif]

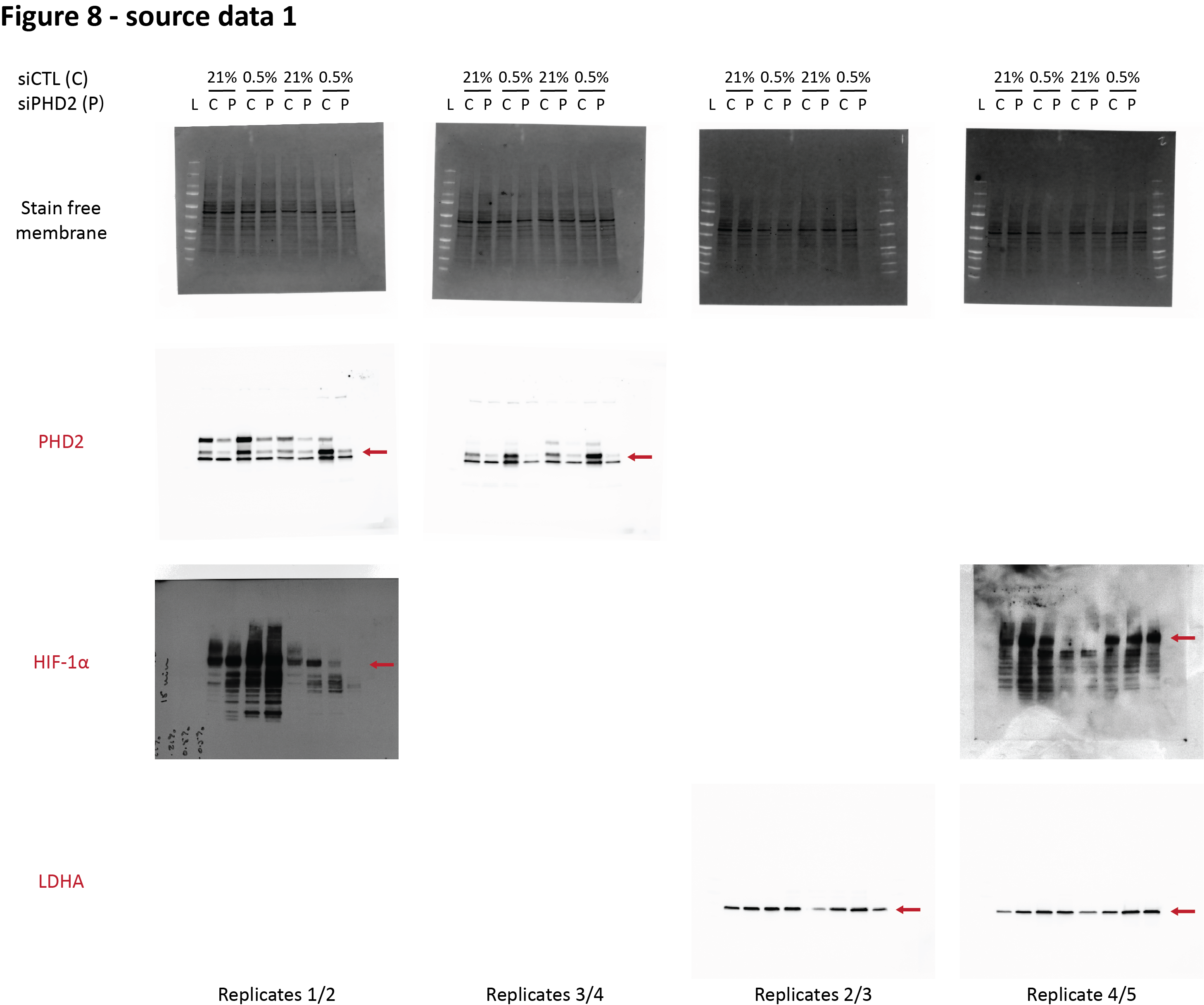

Supplement: Figure 8—figure supplement 1—source data 1. [file elife-82597-fig8-figsupp1-data1.zip › Figure 8 - source data 1.png]

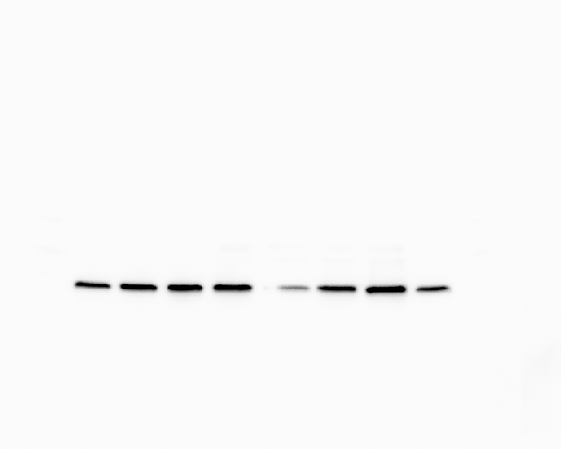

Supplement: Figure 8—figure supplement 1—source data 1. [file elife-82597-fig8-figsupp1-data1.zip › lf_05-siphd_a_ldha_2-3_2023-03-18.tif]

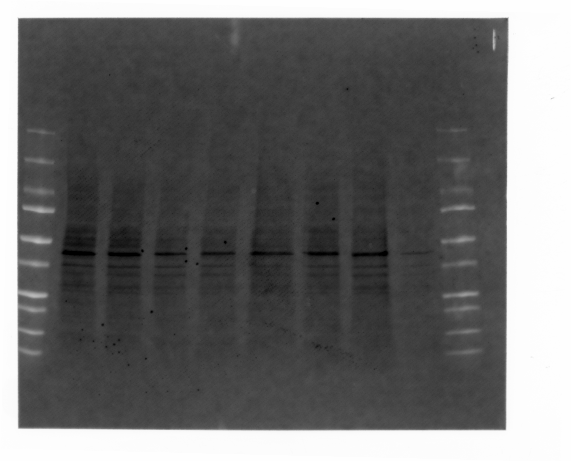

Supplement: Figure 8—figure supplement 1—source data 1. [file elife-82597-fig8-figsupp1-data1.zip › lf_05-siphd_a_membrane_2-3_2023-03-15.tif]

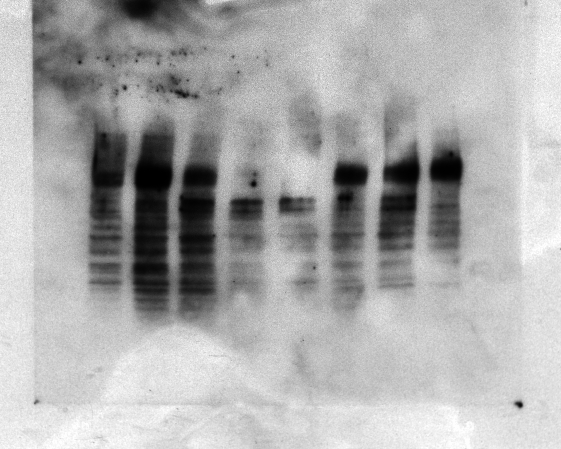

Supplement: Figure 8—figure supplement 1—source data 1. [file elife-82597-fig8-figsupp1-data1.zip › lf_05-siphd_b_hif_4-5_2023-03-15.tif]

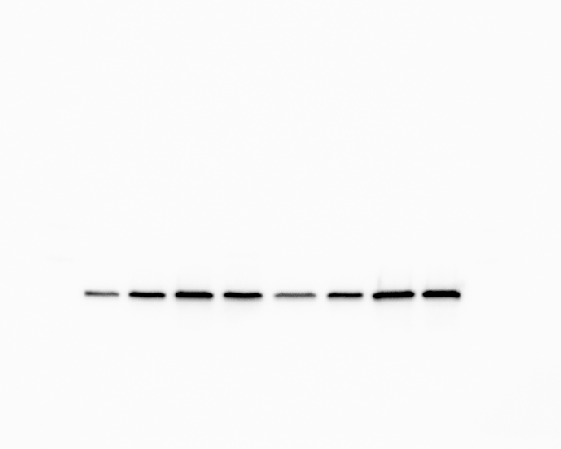

Supplement: Figure 8—figure supplement 1—source data 1. [file elife-82597-fig8-figsupp1-data1.zip › lf_05-siphd_b_ldha_4-5_2023-03-18.tif]

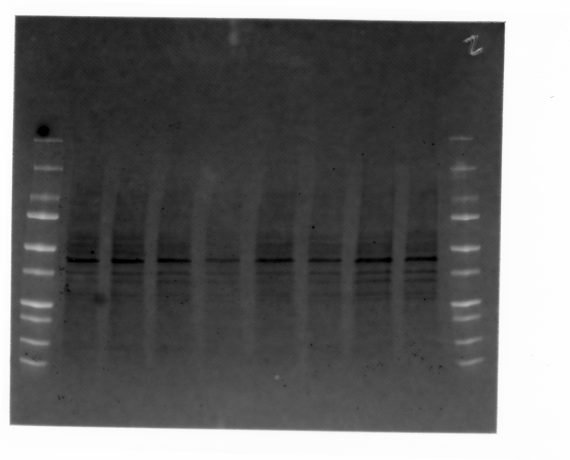

Supplement: Figure 8—figure supplement 1—source data 1. [file elife-82597-fig8-figsupp1-data1.zip › lf_05-siphd_b_membrane_4-5_2023-03-15.tif]

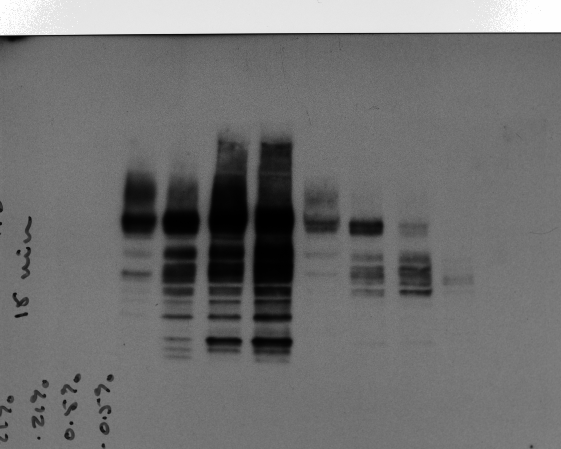

Supplement: Figure 8—figure supplement 1—source data 1. [file elife-82597-fig8-figsupp1-data1.zip › lf_05-siphd_c_hif_1-2_2023-03-23.tif]

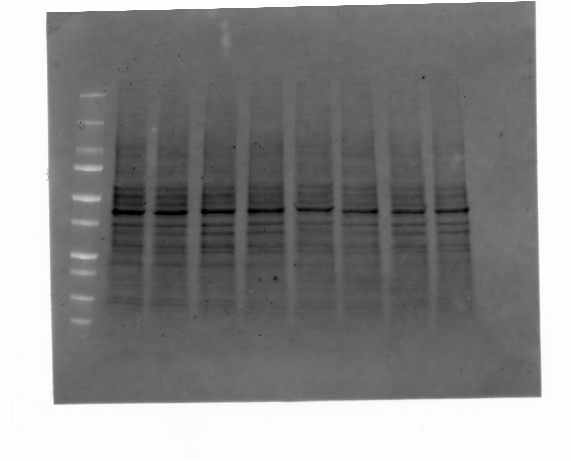

Supplement: Figure 8—figure supplement 1—source data 1. [file elife-82597-fig8-figsupp1-data1.zip › lf_05-siphd_c_membrane_1-2_2023-03-23.tif]

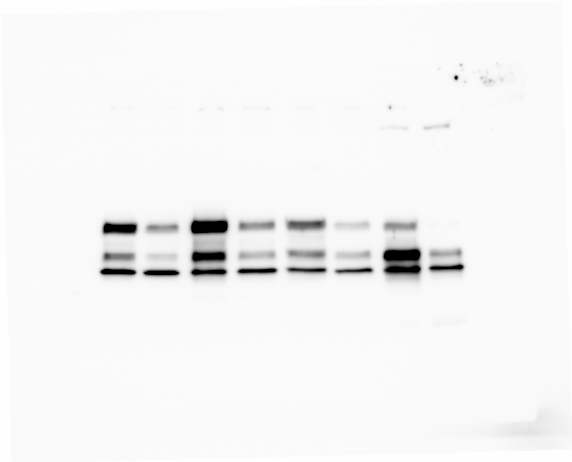

Supplement: Figure 8—figure supplement 1—source data 1. [file elife-82597-fig8-figsupp1-data1.zip › lf_05-siphd_c_phd2_1-2_2023-03-25.tif]

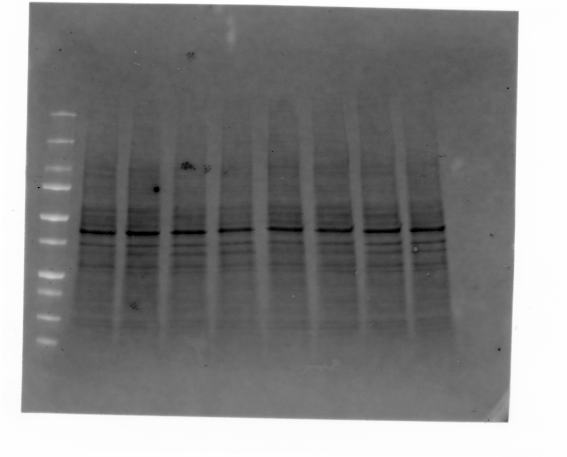

Supplement: Figure 8—figure supplement 1—source data 1. [file elife-82597-fig8-figsupp1-data1.zip › lf_05-siphd_d_membrane_3-4_2023-03-23.tif]

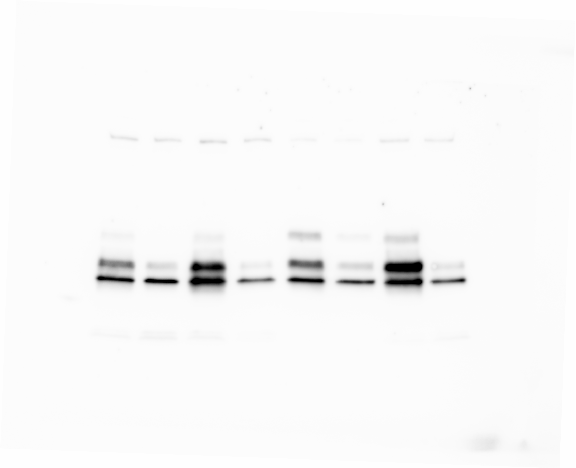

Supplement: Figure 8—figure supplement 1—source data 1. [file elife-82597-fig8-figsupp1-data1.zip › lf_05-siphd_d_phd2_3-4_2023-03-25.tif]

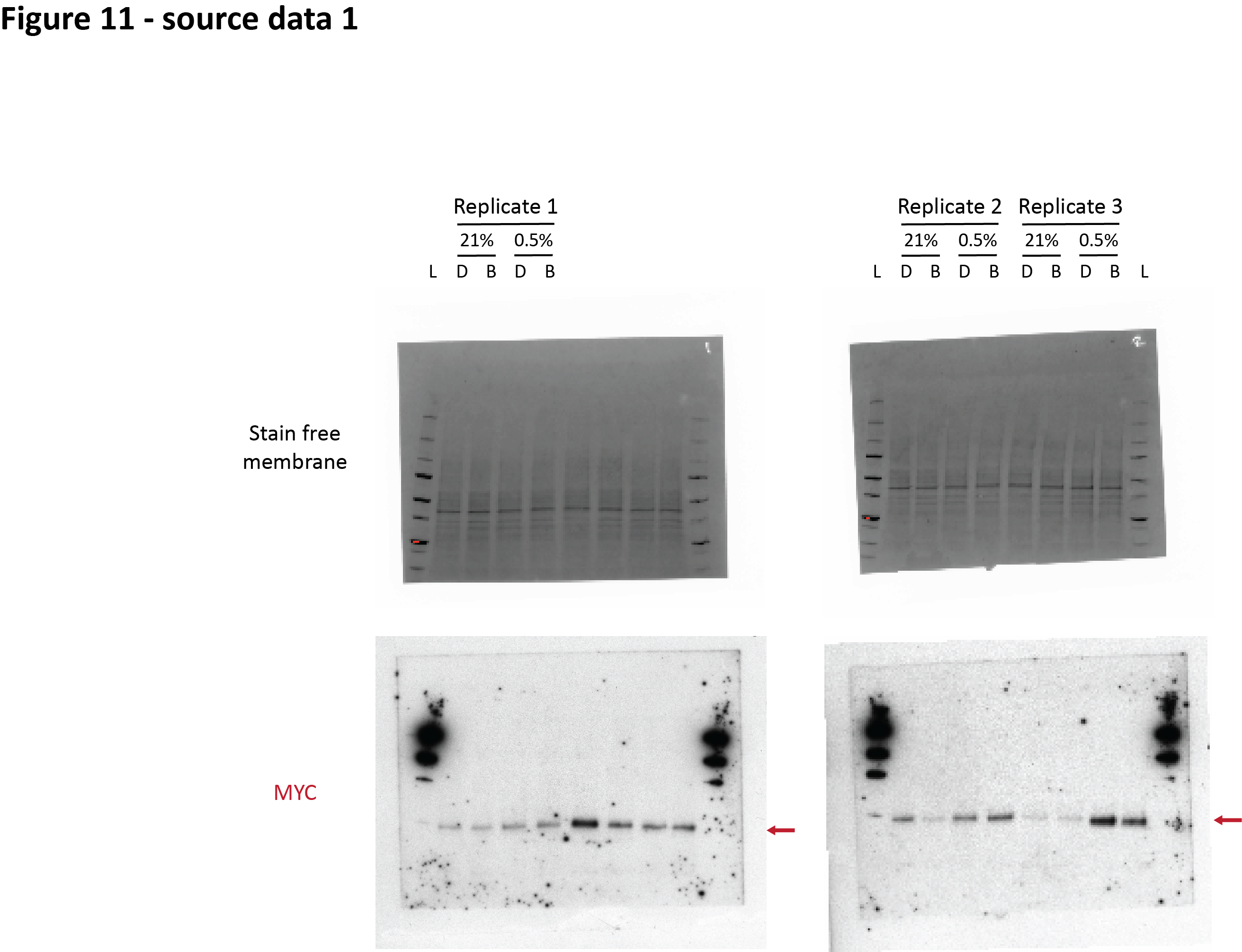

Supplement: Figure 11—source data 1. [file elife-82597-fig11-data1.zip › Figure 11 - source data 1.png]

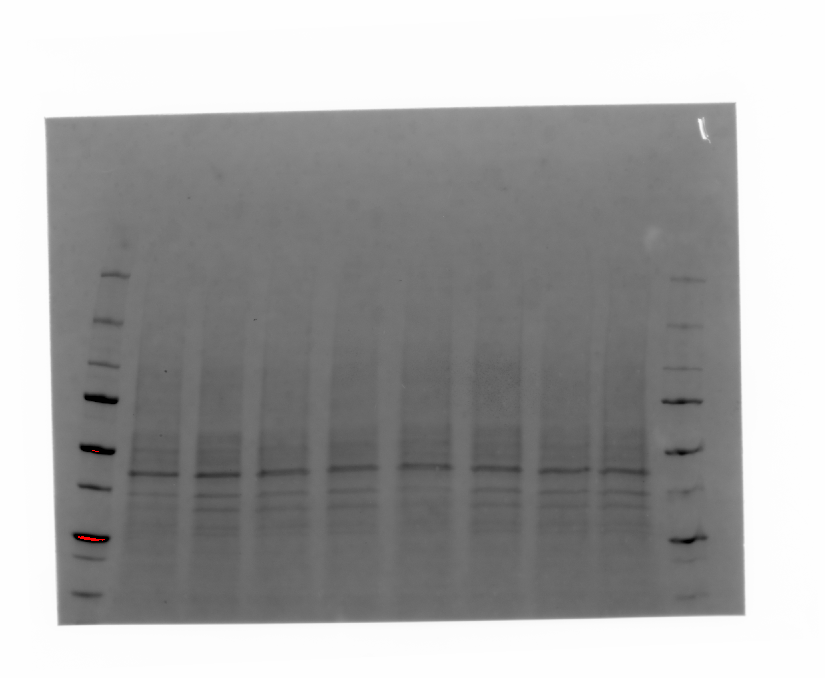

Supplement: Figure 11—source data 1. [file elife-82597-fig11-data1.zip › lf_05-bay_ab_membrane_2021-01-04.tif]

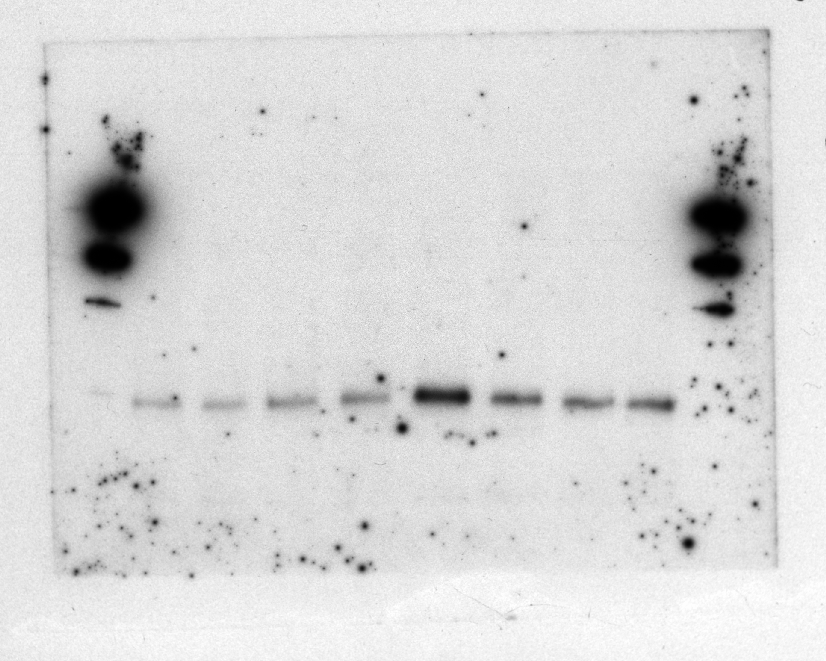

Supplement: Figure 11—source data 1. [file elife-82597-fig11-data1.zip › lf_05-bay_ab_myc_2021-01-06.tif]

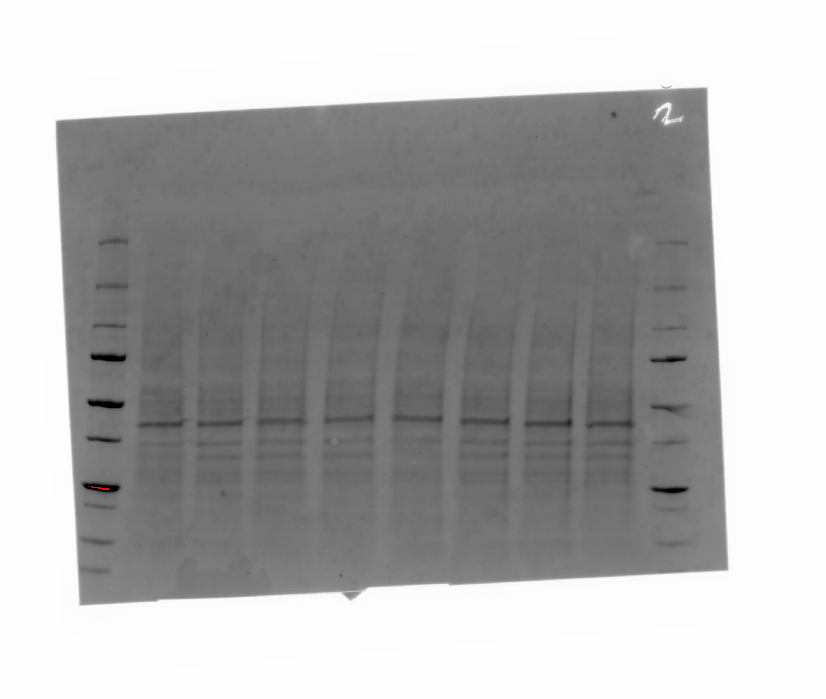

Supplement: Figure 11—source data 1. [file elife-82597-fig11-data1.zip › lf_05-bay_cd_membrane_2021-01-04.tif]

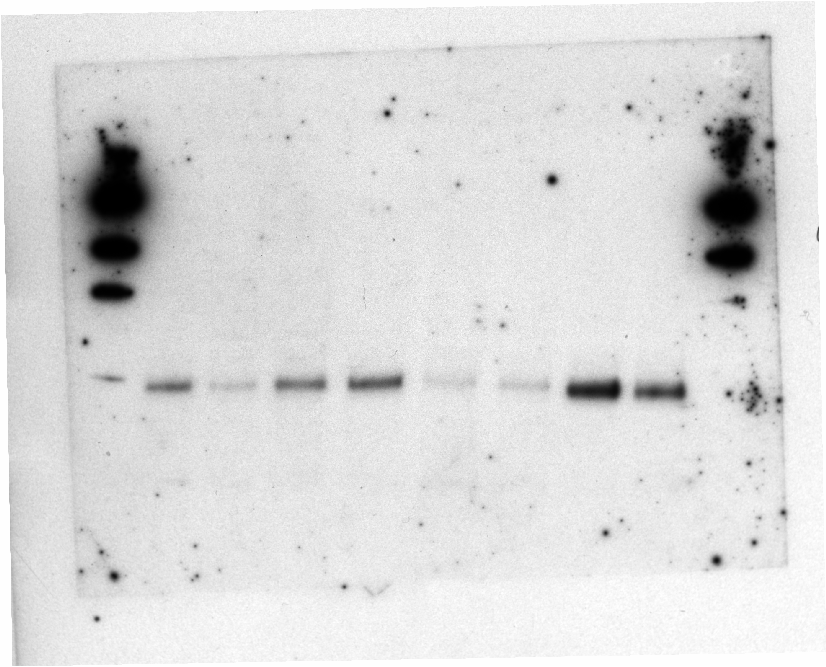

Supplement: Figure 11—source data 1. [file elife-82597-fig11-data1.zip › lf_05-bay_cd_myc_2021-01-06.tif]

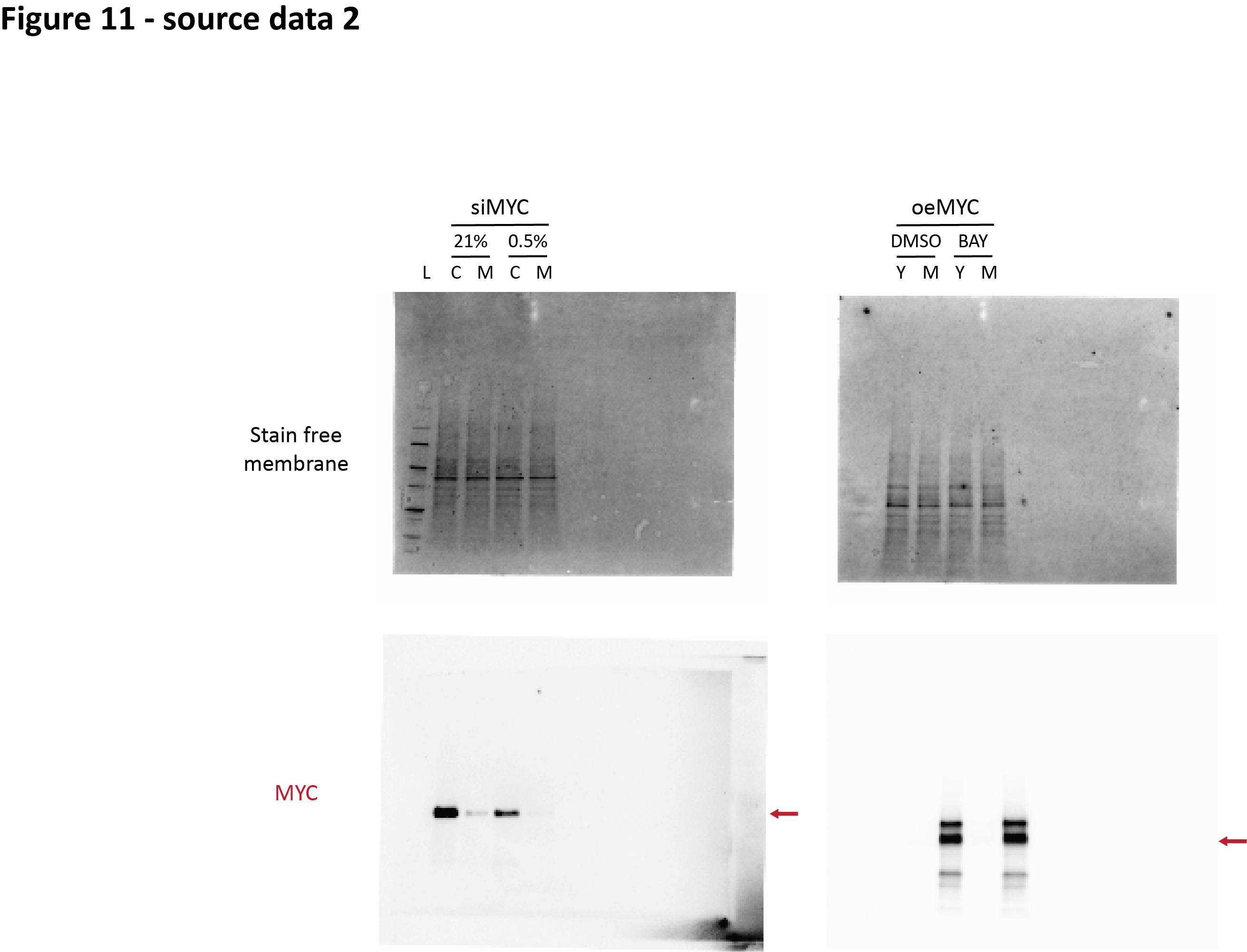

Supplement: Figure 11—source data 2. [file elife-82597-fig11-data2.zip › Figure 11 - source data 2.png]

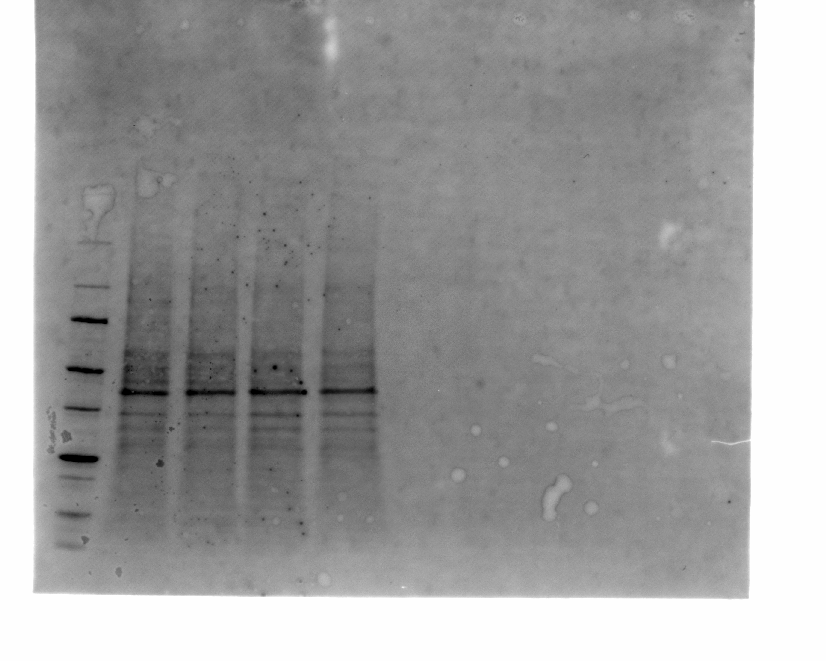

Supplement: Figure 11—source data 2. [file elife-82597-fig11-data2.zip › lf_05-simyc_a_membrane_2021-10-18.tif]

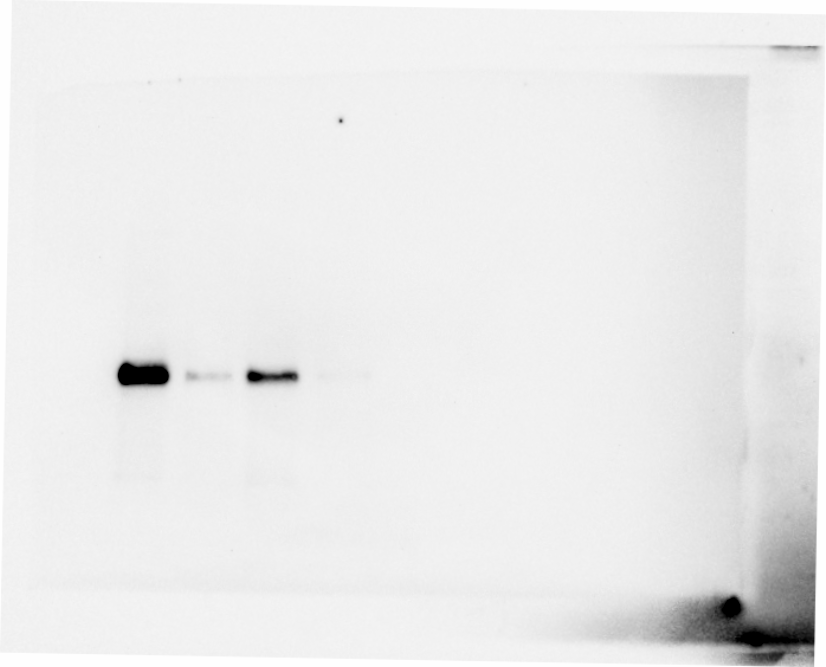

Supplement: Figure 11—source data 2. [file elife-82597-fig11-data2.zip › lf_05-simyc_a_myc_2021-10-19.tif]

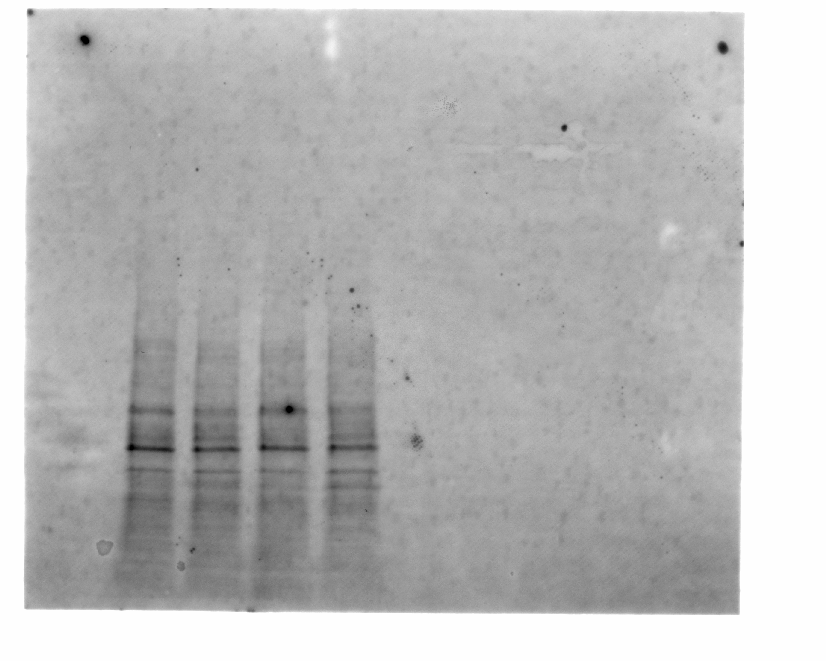

Supplement: Figure 11—source data 2. [file elife-82597-fig11-data2.zip › lf_bay-myc_b_membrane_2021-09-27.tif]

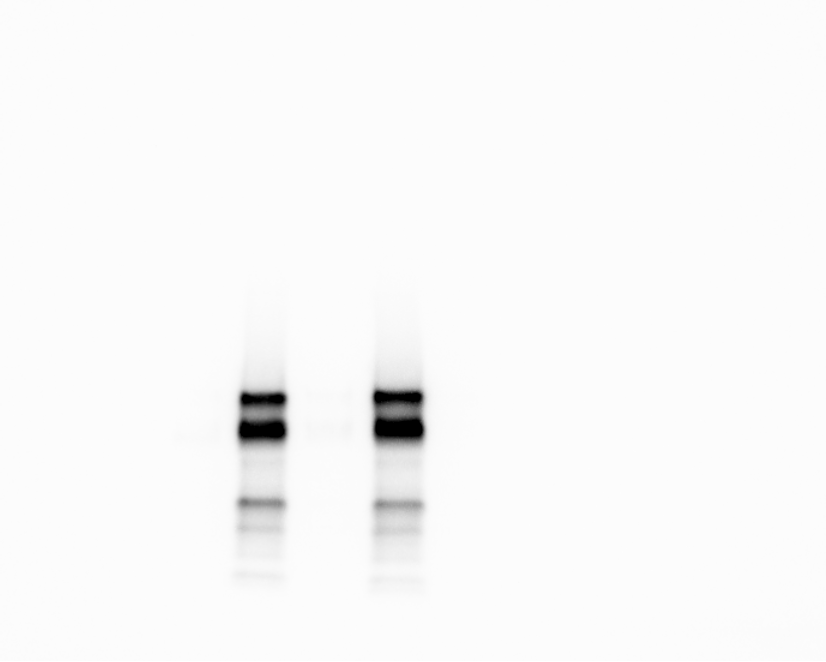

Supplement: Figure 11—source data 2. [file elife-82597-fig11-data2.zip › lf_bay-myc_b_myc_2021-09-28.tif]

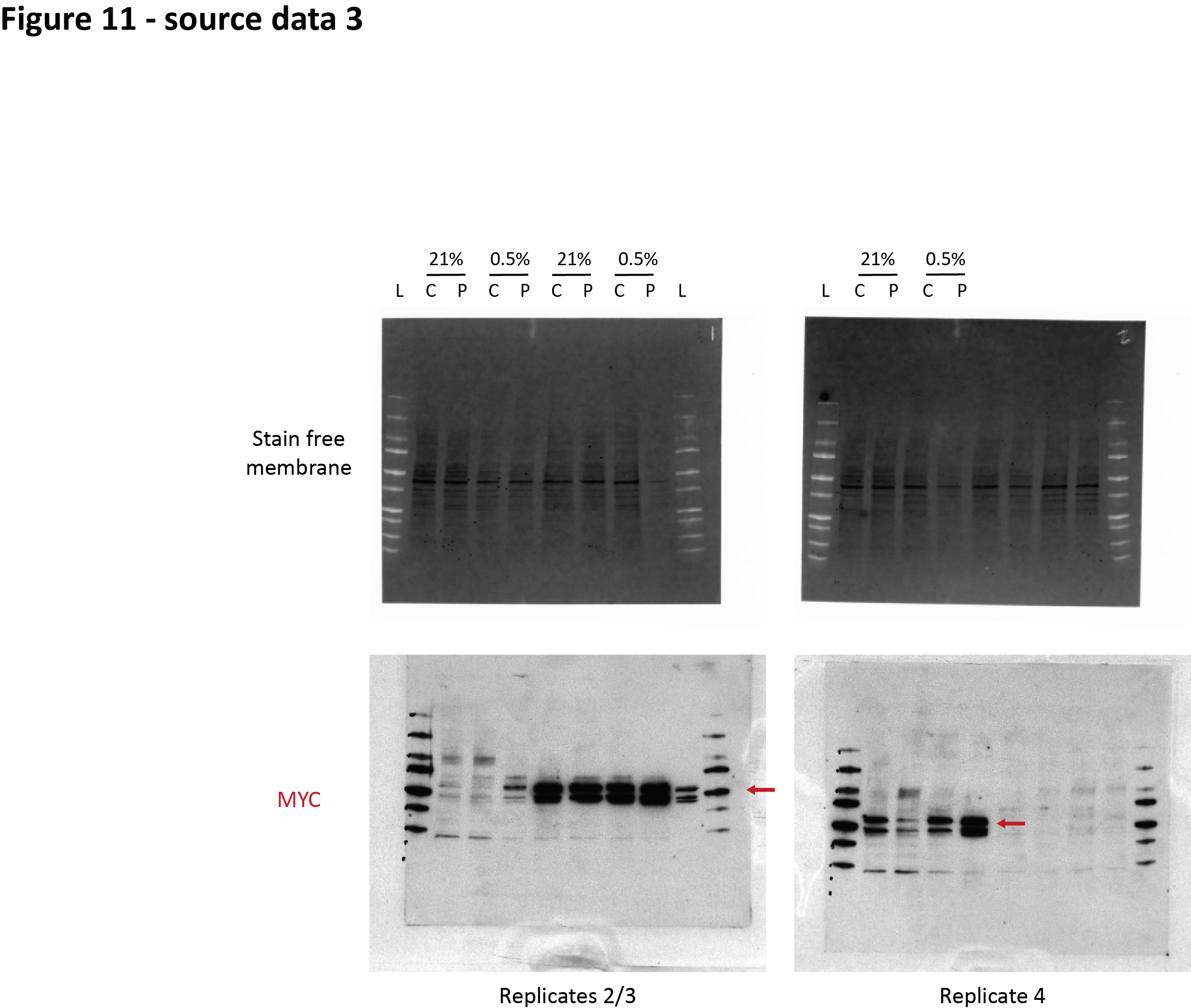

Supplement: Figure 11—figure supplement 1—source data 1. [file elife-82597-fig11-figsupp1-data1.zip › Figure 11 - source data 3.png]

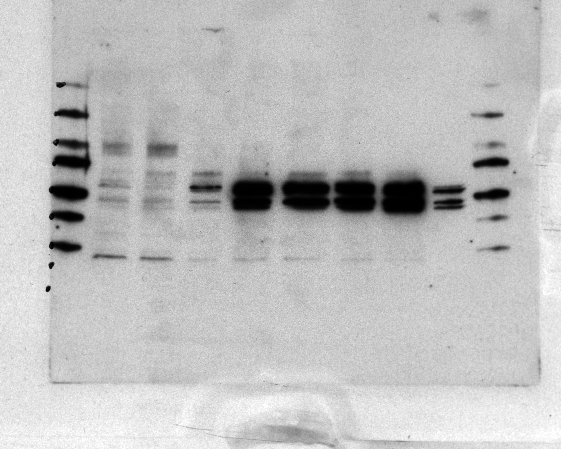

Supplement: Figure 11—figure supplement 1—source data 1. [file elife-82597-fig11-figsupp1-data1.zip › lf_05-siphd_a_myc_2-3_2023-03-15.tif]

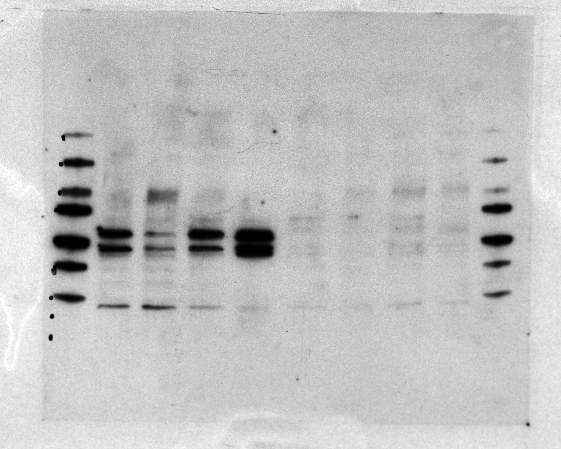

Supplement: Figure 11—figure supplement 1—source data 1. [file elife-82597-fig11-figsupp1-data1.zip › lf_05-siphd_b_myc_4-5_2023-03-15.tif]

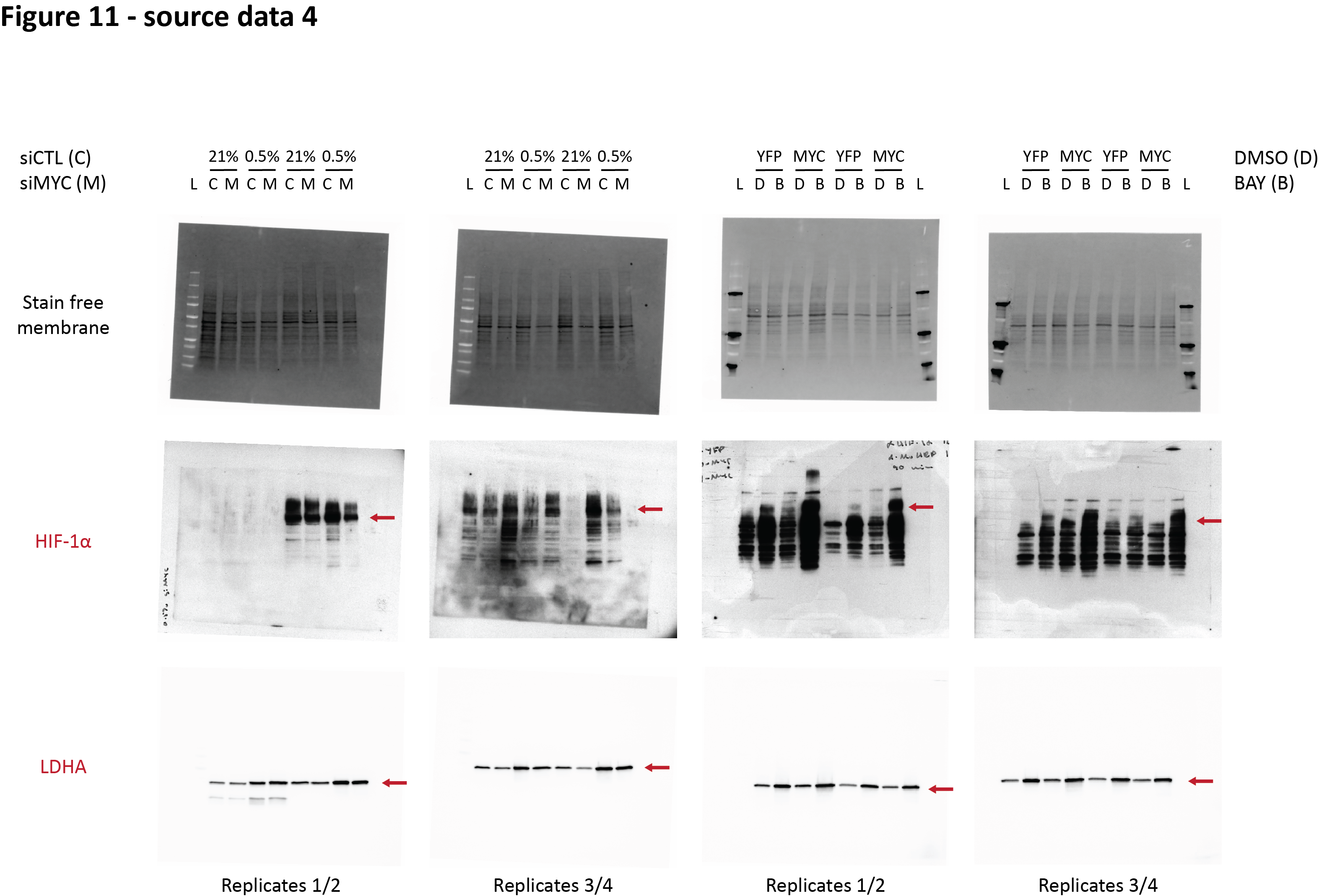

Supplement: Figure 11—figure supplement 2—source data 1. [file elife-82597-fig11-figsupp2-data1.zip › Figure 11 - source data 4.png]

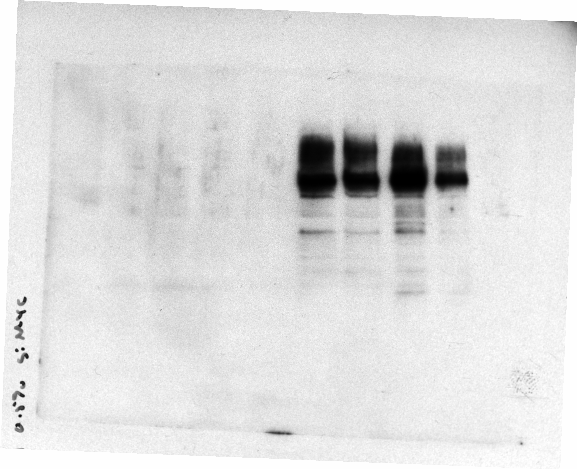

Supplement: Figure 11—figure supplement 2—source data 1. [file elife-82597-fig11-figsupp2-data1.zip › lf_05-simyc_b_hif_2023-03-21.tif]

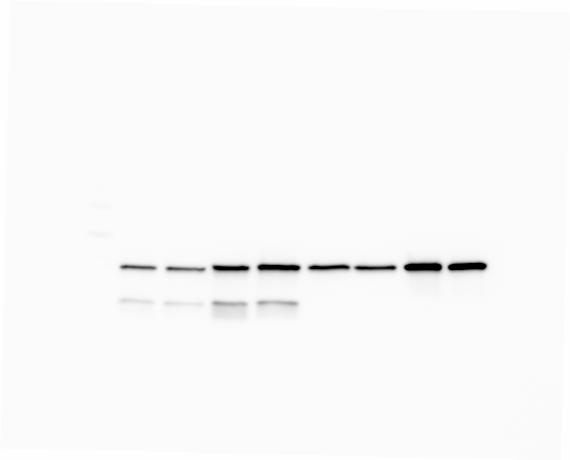

Supplement: Figure 11—figure supplement 2—source data 1. [file elife-82597-fig11-figsupp2-data1.zip › lf_05-simyc_b_ldha_2023-03-24.tif]

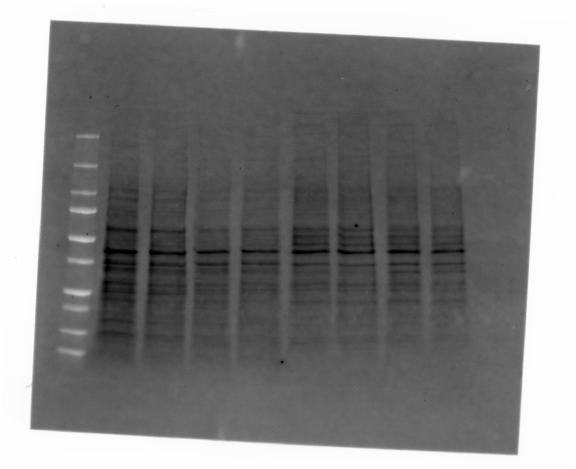

Supplement: Figure 11—figure supplement 2—source data 1. [file elife-82597-fig11-figsupp2-data1.zip › lf_05-simyc_b_membrane_2023-03-21.tif]

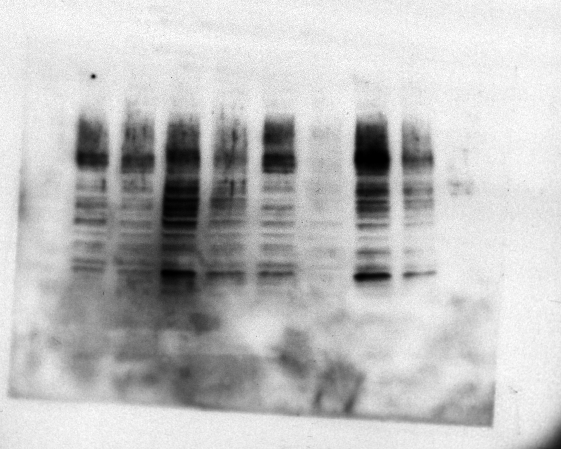

Supplement: Figure 11—figure supplement 2—source data 1. [file elife-82597-fig11-figsupp2-data1.zip › lf_05-simyc_c_hif_2023-03-21.tif]

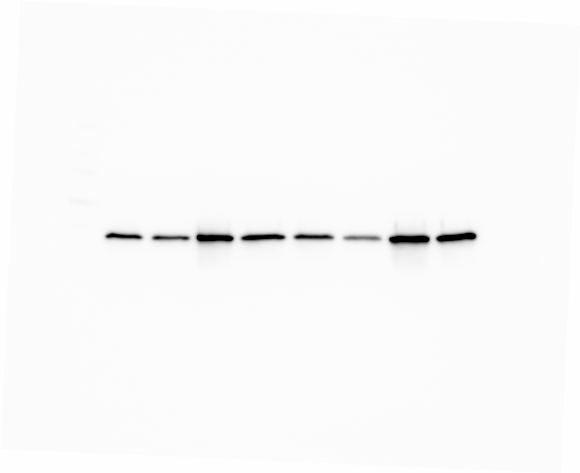

Supplement: Figure 11—figure supplement 2—source data 1. [file elife-82597-fig11-figsupp2-data1.zip › lf_05-simyc_c_ldha_2023-03-24.tif]

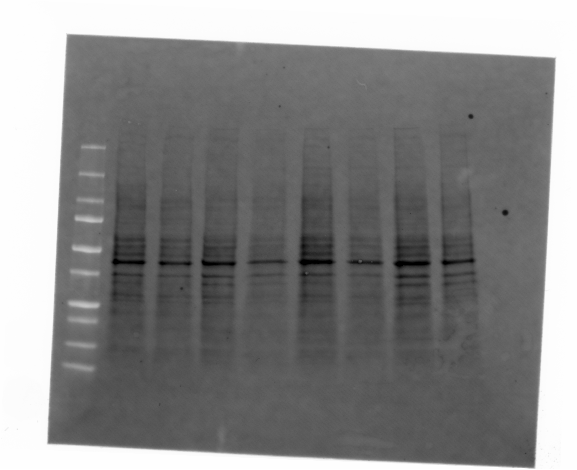

Supplement: Figure 11—figure supplement 2—source data 1. [file elife-82597-fig11-figsupp2-data1.zip › lf_05-simyc_c_membrane_2023-03-21.tif]

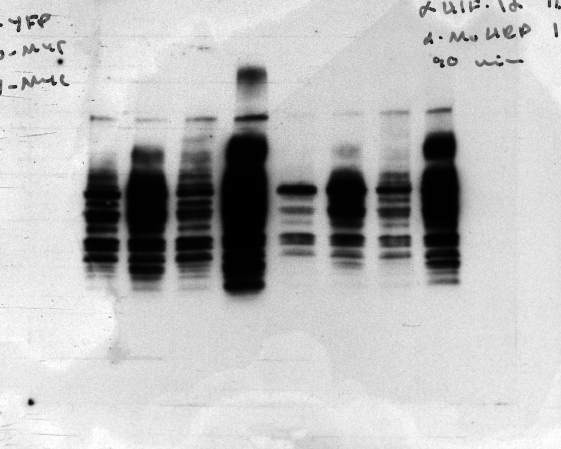

Supplement: Figure 11—figure supplement 2—source data 1. [file elife-82597-fig11-figsupp2-data1.zip › lf_bay-myc_c_hif_2023-03-13.tif]

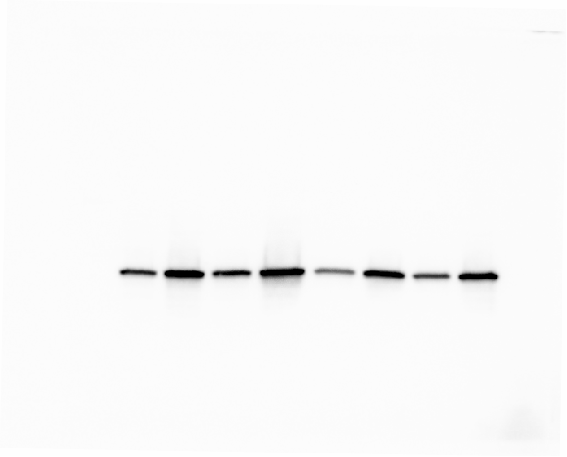

Supplement: Figure 11—figure supplement 2—source data 1. [file elife-82597-fig11-figsupp2-data1.zip › lf_bay-myc_c_ldha_2023-03-15.tif]

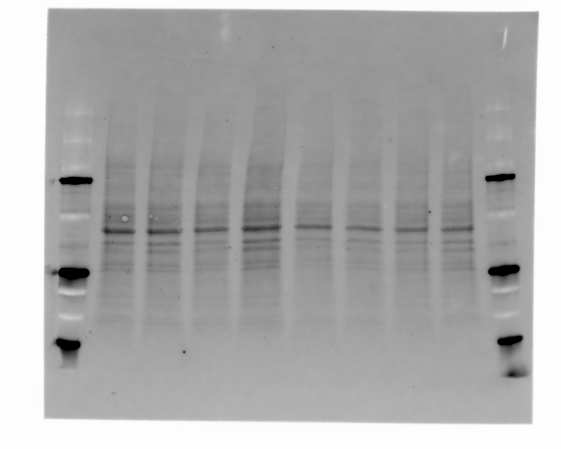

Supplement: Figure 11—figure supplement 2—source data 1. [file elife-82597-fig11-figsupp2-data1.zip › lf_bay-myc_c_membrane_2023-03-13.tif]

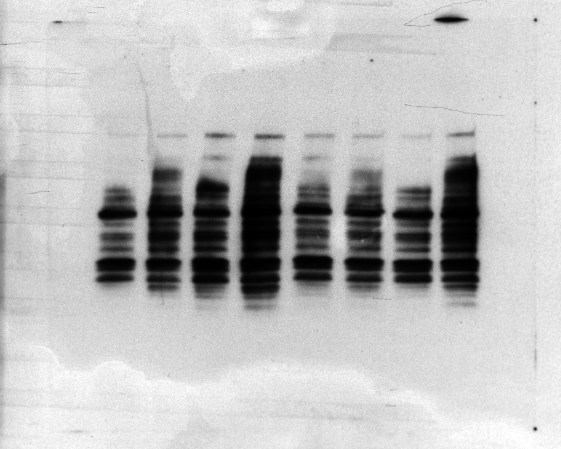

Supplement: Figure 11—figure supplement 2—source data 1. [file elife-82597-fig11-figsupp2-data1.zip › lf_bay-myc_d_hif_2023-03-13.tif]

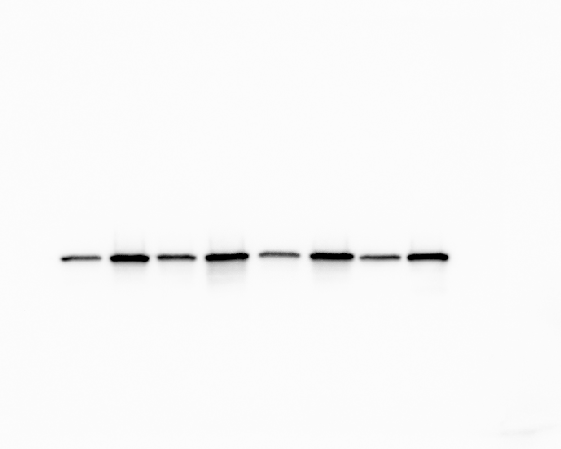

Supplement: Figure 11—figure supplement 2—source data 1. [file elife-82597-fig11-figsupp2-data1.zip › lf_bay-myc_d_ldha_2023-03-15.tif]

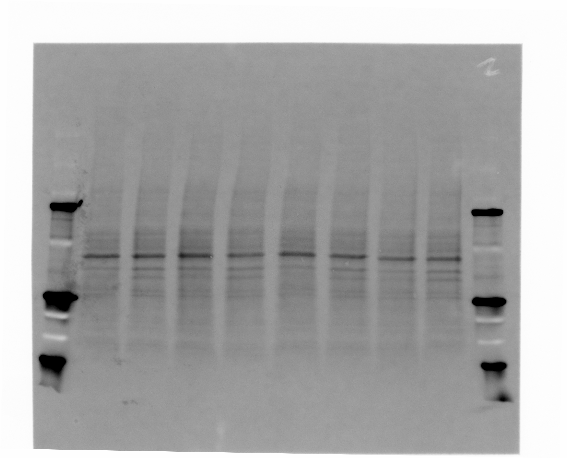

Supplement: Figure 11—figure supplement 2—source data 1. [file elife-82597-fig11-figsupp2-data1.zip › lf_bay-myc_d_membrane_2023-03-13.tif]
